# The ECF sigma factor, PSPTO_1043, in *Pseudomonas syringae* pv. *tomato* DC3000 is induced by oxidative stress and regulates genes involved in oxidative stress response

**DOI:** 10.1371/journal.pone.0180340

**Published:** 2017-07-12

**Authors:** Bronwyn G. Butcher, Zhongmeng Bao, Janet Wilson, Paul Stodghill, Bryan Swingle, Melanie Filiatrault, David Schneider, Samuel Cartinhour

**Affiliations:** 1 School of Integrative Plant Science, Section of Plant Pathology and Plant-Microbe Biology, Cornell University, Ithaca, NY 14853, United States of America; 2 Robert W. Holley Center, USDA-ARS, 583 Tower Road, Ithaca, NY 14853, United States of America; Centre National de la Recherche Scientifique, Aix-Marseille Université, FRANCE

## Abstract

The bacterial plant pathogen *Pseudomonas syringae* adapts to changes in the environment by modifying its gene expression profile. In many cases, the response is mediated by the activation of extracytoplasmic function (ECF) sigma factors that direct RNA polymerase to transcribe specific sets of genes. In this study we focus on PSPTO_1043, one of ten ECF sigma factors in *P. syringae* pv. *tomato* DC3000 (DC3000). PSPTO_1043, together with PSPTO_1042, encode an RpoE_Rsp_/ChrR-like sigma/anti-sigma factor pair. Although this gene pair is unique to the *P. syringae* group among the pseudomonads, homologous genes can be found in photosynthetic genera such as *Rhodospirillum*, *Thalassospira*, *Phaeospirillum* and *Parvibaculum*. Using ChIP-Seq, we detected 137 putative PSPTO_1043 binding sites and identified a likely promoter motif. We characterized 13 promoter candidates, six of which regulate genes that appear to be found only in *P. syringae*. PSPTO_1043 responds to the presence of singlet oxygen (^1^O_2_) and tert-butyl hydroperoxide (tBOOH) and several of the genes regulated by PSPTO_1043 appear to be involved in response to oxidative stress.

## Introduction

*Pseudomonas syringae* is a diverse species complex with more than 50 described pathovars causing speck, fleck, spot, blight and canker diseases on a wide range of hosts [1]. However, individual isolates typically have a narrow host range. The organism is often present as an epiphyte but can enter the plant via stomata or wound sites, proliferate in intercellular spaces, and eventually cause disease. *P. syringae* pv. *tomato* DC3000 (DC3000) [2] causes bacterial speck on tomato plants and *Arabidopsis thaliana* and is a model organism for the study of plant pathogen interactions (reviewed in [3]).

Bacteria employ diverse strategies for responding to stressful changes in the environment. One important mechanism is mediated by extracytoplasmic function (ECF) sigma factors [4], which are typically co-transcribed with anti-sigma factors that limit their activity until a specific signal occurs. The sigma factor is released from or is activated by the anti-sigma factor and directs RNA polymerase to express genes that are required by the cell to adapt to the new environment. A subset of genes regulated in this fashion is referred to as a regulon.

The DC3000 genome encodes ten ECF-type sigma factors [5], five of which are FecI-type iron responsive sigma factors [6–8]. The remaining five ECF sigma factors include HrpL, which activates expression of the Hrp/Hrc type III secretion system, various effector proteins [9–11], and AlgU, which is responsible for regulating genes involved in alginate production [12] and components of the Hrp type III secretion system [13].

Here we present an analysis of PSPTO_1043 and PSPTO_1042. We show that these two genes are homologous to RpoE_Rsp_ (RSP1093) and ChrR(RSP1092), a sigma factor/anti-sigma factor regulatory system found in *Rhodobacter sphaeroides*, and similar genes in other bacteria. We use the “Rsp” subscript to reinforce that we are referring to the gene in *R. sphaeroides* and not the well-studied gene with the same name (*σ*^*E*^) in *E. coli*. RpoE_Rsp_/ChrR is [14] involved with response to singlet oxygen (^1^O_2_) in *R. sphaeroides*. In this paper, we show that the PSPTO_1043/1042 system in DC3000 responds to the presence of ^1^O_2_, produced by Rose Bengal, and tert-butyl hydroperoxide (tBOOH). These conditions were chosen to stimulate an oxidative stress response, although the precise identity of the signaling species have not been determined.

## Methods

### Bacterial strains and growth conditions

The strains, plasmids and primers used for this study are listed in S1 Table. DC3000 was cultured in King’s B medium (KB) [15] or modified Luria broth (LM) [16] at 28°C. Our formulation of LM is (per liter) 10g Bacto tryptone, 6g yeast extract, 0.6g NaCl, 0.4g MgSO_4_⋅7H_2_O, and 1.5g K_2_HPO_4_.

### Construction of strains overexpressing PSPTO_1043

The PSPTO_1043 coding region was amplified with primers oSWC01724 and oSWC01725 (S1 Table) using the Expand High Fidelity PCR System (Roche). PCR fragments were gel purified using the Zymoclean Gel DNA Recovery Kit (Zymo Research) and cloned into pENTR/SD/D (Invitrogen) by directional TOPO cloning to create pBB36. The pBB45 expression construct (where PSPTO_1043-FLAG is expressed under control of the constitutive *nptII* promoter) was constructed by performing an LR reaction with the pBS46 expression vector [6] using LR Clonase II Enzyme Mix (Invitrogen). The resulting plasmid was sequenced to confirm structure and was then transformed into DC3000 by electroporation to create strain BBPS21.

A strain overexpressing wild-type (WT) (untagged) PSPTO_1043 was similarly constructed using primers oSWC07 and oSWC08 (S1 Table) creating an entry plasmid (pBS5), which was used to construct the overexpression plasmid, pBS163. This was transformed into DC3000 by electroporation creating BBPS55.

### ChIP-Seq

The *P. syringae* PSPTO_1043-FLAG expression strain (BBPS21) was grown overnight at 28°C in LM medium containing 5 *μ*g/ml gentamicin and was used to inoculate 100 ml LM medium at an optical density at 600nm (OD_600_) of approximately 0.05. Cells were grown with shaking at 28°C to mid-log (OD_600_ approximately 0.5). Expression of the FLAG tagged PSPTO_1043 under these conditions was confirmed by Western blotting with monoclonal anti-FLAG M2-alkaline phosphatase antibody (Sigma-Aldrich, St. Louis, MO).

Chromatin immunoprecipitation was performed as previously described [7]. Briefly, cell proteins were cross-linked by adding formaldehyde at 1% concentration to the medium. After quenching the reaction with 0.36 mM glycine, the cells were lysed and sonication was used to shear the DNA to fragments with an average approximate length of 400bp. Centrifugation was used to extract supernatant fluid, a portion of which was set aside as the lysate control. Commercially available anti-FLAG biotinylated M2 antibodies (Sigma-Aldrich, St. Louis, MO) were used to isolate the DNA-protein complexes containing the FLAG-tagged sigma factors from the remaining supernatant. The isolated complexes were used as the IP samples. The DNA for both the lysate and IP samples was purified by reversing the cross-link reaction, applying pronase (Sigma-Aldrich, St. Louis, MO) to digest the protein, and QIAquick PCR purification (Qiagen, Hilden, Germany).

The purified DNA was subjected to library preparation and high throughput sequencing using the Illumina GA II platform (Illumina, San Diego, CA) by the Cornell University Life Sciences Core Laboratories Center. The FASTQ files returned by the sequencing facility were deposited in NCBI’s Sequence Read Archive (#SRR1583166).

### Analysis of ChIP-Seq data

The sequenced reads were used to construct genomic profiles following the method described in [17]. Version 2.20 of the SOAP2 alignment package [18] was used. Regions enriched by immunoprecipitation, here referred to as peaks, were identified computationally using CSDeconv [19] version 1.02, as described in [20] (with parameters llr = 3 and alpha = 1000). Several peaks fell within the PSPTO_1043 gene. Since these were probably artifacts due to DNA recovered from the multicopy PSPTO_1043 overexpression plasmid, they were removed from the list of identified peak regions. Next, sequences that fell within each identified peak region or within 50 bps of either side of the peak boundary were extracted, and motif discovery was performed using MEME [21] 4.10.0 (patch 1) with the parameters,

-revcomp -minw 18 -maxw 40 -nmotifs 5 -mod zoops -maxsites 137 -dna

### RNA isolation and real-time PCR (RT-PCR)

DC3000 cells overexpressing either PSPTO_1043 or FLAG-tagged PSPTO_1043, along with DC3000 containing the empty vector, pBS60, were grown under conditions identical to those used for the ChIP-Seq experiments. Samples (1 mL) were withdrawn from each culture and centrifuged to obtain cell pellets. Pellets were stored at -80°C. RNA was isolated from cells using the Qiagen RNeasy kit as previously described [22]. Real-time PCR was performed as previously described [22] and changes in expression between the PSPTO_1043 overexpressing strains versus those carrying the empty vector control were calculated using the ΔΔCt method (ΔCt_sample_ - ΔCt_reference_) where the housekeeping gene, *gap-1* (PSPTO_1287), is used as the reference.

### Creation of PSPTO_1043/1042 double mutant

The DC3000 ΔPSPTO_1043/1042 double mutant strain was constructed using marker exchange mutagenesis as described in [23]. Regions flanking the PSPTO_1043/1042 locus were amplified by PCR using the primer pairs oSWC2022/2024 and oSWC2025/2026 (S1 Table) with DC3000 genomic DNA as template to generate 1.1 and 1.0-kb products corresponding to the regions upstream of PSPTO_1042 and downstream of PSPTO_1043, respectively. Products were gel purified and used as templates in a SOEing PCR reaction [24] with oSWC2023/2027 (S1 Table), which joined the PSPTO_1043/1042 flanks and introduced XmaI sites at both ends of the product. The approximately 2.0 kb product was gel purified, digested with XmaI and ligated to pK18mobsacB [25] cut with the same restriction enzyme to produce pZB30. The structure of the pZB30 plasmid insert was confirmed by restriction digest and Sanger sequencing. The pZB30 deletion construct was introduced into DC3000 by electroporation and plasmid integration events were selected on KB medium containing 50 *μ*g/ml kanamycin. Clones that had subsequently lost the pK18mobSacB sequences containing *sacB* were selected on medium containing 10% sucrose. Sucrose resistant ΔPSPTO_1043/1042 clones were screened by PCR and positive clones were confirmed by sequencing.

### Creation of *lux* fusions with putative PSPTO_1043 controlled promoters

Predicted promoter regions upstream of PSPTO_1043, *phrB* (PSPTO_1121), *katG* (PSPTO_4530) and PSPTO_1900 were amplified with primers shown in S1 Table using Premix Ex Taq (Takara). The PCR products were purified using the DNA Clean and Concentrator kit (Zymo Research) and cloned using the pENTR/D-TOPO cloning kit (Invitrogen) to generate entry clones pBB56, pBB57, pBB58 and pBB59, respectively. The *lux* fusions were created by LR reaction between the entry clones and a destination vector, pBS58 [6], using LR clonase II (Invitrogen). The resulting plasmids were sequenced to confirm structure and transformed by electroporation into DC3000, BBPS32, BBPS21, BBPS55, and BBPS12, creating strains shown in S1 Table.

### Expression of genes in response to PSPTO_1043 overexpression

Strains carrying the promoter fusions (see above) as well as the plasmid overexpressing PSPTO_1043 (pBB45) or the empty vector (pBS60 [6]) were grown overnight in LM with the appropriate antibiotics at 28°C. Each strain was diluted to an OD_600_ of 0.3 in fresh LM and 200*μ*l of culture was aliquoted into three wells of a 96 well plate, and incubated at room temperature for 6 hrs. Luminescence was measured with a Tecan GENios microplate reader, using Magellan Data Analysis software. Relative luminescence was calculated as luminescence/OD_600_. Technical replicates were averaged, and each experiment was performed three times.

### Growth and expression in presence of ^1^O_2_

^1^O_2_ was generated using the photosensitizer Rose Bengal. Overnight cultures of strains carrying the promoter fusions (grown in LM) were diluted to OD_600_ of approximately 0.2 and 200*μ*l aliquoted into wells of a 96 well plate. Rose Bengal at a final concentration of 2.5*μ*M was added to the wells. Half of the plate was covered with adhesive foil seal (over the lid of the plate) to create “dark” conditions in which little or no ^1^O_2_ is produced. Wells without Rose Bengal (in both the light and dark sections of the plate) were used as controls. The plate was then incubated with shaking at room temperature in the light and OD_600_ and luminescence measured every 30 minutes using a Synergy 2 Microplate reader (Biotek). Relative luminescence was calculated as luminescence/OD_600_. Technical replicates were averaged, and each experiment was performed three times. We confirmed production of ^1^O_2_ under these conditions using 5 *μ*M of the ^1^O_2_ sensor green reagent (Molecular probes, Life technologies) and measuring fluorescence in the Biotek plate reader using excitation/emission wavelengths of 485/516 nm.

### Growth and expression in presence of tBOOH

Cultures of DC3000 and mutant derivatives grown overnight in LM were diluted to OD_600_ of approximately 0.1 in fresh LM and 200*μ*l aliquoted into wells of 96 a well plate. tert-Butyl hydroperoxide (tBOOH) at a final concentration of 0.1mM was added to the wells. The plate was then incubated at 28°C with shaking and OD_600_ and luminescence measured every 1 hour using a Biotek Synery Microbplate reader (Biotek). Relative luminescence was calculated as luminescence/OD_600_. Technical replicates were averaged, and each experiment was performed three times.

## Results

### PSPTO_1043/1042 are specific to the *P. syringae* group of pseudomonads

PSPTO_1043 was previously described as encoding a *P. syringae* specific sigma factor [5] after a survey of a small number of *Pseudomonas* genome sequences. Because many more complete and partially-sequenced genomes became available, we reexamined the distribution of the PSPTO_1043 sigma factor and its cognate anti-sigma factor (PSPTO_1042) genes by aligning their predicted amino acid sequences against the non-redundant protein sequences (NR) database using NCBI’s BLAST server [26]. Homologs to both were identified in *P. syringae* strains. as well as unrelated *Rhodanobacter* species and *Stenotrophomonas maltophilia* (Table 1A). The later two are naturally found in soil and subsurface environments and *S. maltophilia* is also a common nosocomial multi-drug-resistant pathogen in immune-compromised patients. More distant homologs of the PSPTO_1043/1042 proteins are also found in many photosynthetic bacteria species such as those in the *Rhodospirillum*, *Thalassospira*, *Phaeospirillum* and *Parvibaculum* genera (Table 1A).

**Table 1:** Distribution of PSPTO_1043/1042 orthologs.

A. Summary of BLAST results		
Bacterial strains	PSPTO_1043% Identity	PSPTO_1042% Identity
*P. syringae* strains	92-100%	82-100%
*Rhodanobacter* and *Stenotrophomonas*	49-54%	42-52%
Photosynthetic bacteria	<45%	<38%
B. Results for several non-*Pseudomonas* species		
Bacterial strains	PSPTO_1043% Identity	PSPTO_1042% Identity
*Caulobacter crescentus*	40%	32%
*Rhodobacter sphaeroides*	38%	35%
*Azospirillum bracilense*	40%	33%
*Roseobacter denitrificans*	39%	34%

Examining some of these species more closely, PSPTO_1043 and PSPTO_1042 showed 32-40% identity to the RpoE_Rsp_-ChrR systems in the bacteria *Rhodobacter sphaeroides* [14], *Caulobacter crescentus* [27], *Azospirillum brasilense* [28] and *Roseobacter denitrificans* [29] (Table 1B). These systems respond to ^1^O_2_ and the corresponding regulons in all but *Azospirillum* have been studied.

In order to test whether the PSPTO_1043/1042 system is unique within the Pseudomonadales to the *P. syringae* species group, we performed a systematic comparative analysis of 1028 closed and draft Pseudomonadales genomes downloaded from GenBank (S2 Table). While genes with sequence similarity to PSPTO_1043 were found in many bacteria (including all *Pseudomonas aeruginosa* strains used in this analysis), the homologs to PSPTO_1042 were found primarily in *P. syringae* strains and other closely related species (*P. avellanae*, *P. savastanoi* and *P. viridiflava*).

### Identification of PSPTO_1043 binding sites

ECF sigma factor activity usually requires a specific signal that results in release of the sigma factor from its anti-sigma factor. Since the identity of the signal for the PSPTO_1043/1042 system was not yet known in DC3000, we expressed a FLAG tagged version of the sigma factor from a constitutive *nptII* promoter on a multicopy plasmid and performed ChIP-Seq to identify putative targets. Cells were collected at the mid-log phase of growth. Following immunoprecipitation, samples were subjected to high throughput sequencing and the sequence data aligned to the genome as described in the Methods. The “sinister” and “naive” profiles of the sequence reads aligned to the DC3000 chromosome (NC_004578.1) can be found in S1 and S2 Datasets respectively.

Regions with an large number of reads, or “peaks”, were readily observed by examining the genomic profiles using the Artemis genome browser [30], as illustrated in S1 Fig. As described in the Methods, we used CSDeconv to computationally identify peaks. 142 peaks were identified. Five peaks fell within the PSPTO_1043 gene. Because these signals are probably artifacts due to DNA recovered from the multicopy PSPTO_1043 overexpression plasmid, they were removed. The remaining 137 peaks (S3 Dataset) were examined further.

As described in the Methods, MEME [21] was used to perform a motif-discovery analysis on the sequences associated with them. Two such regions are shown in S1 Fig.

**Fig 1:**
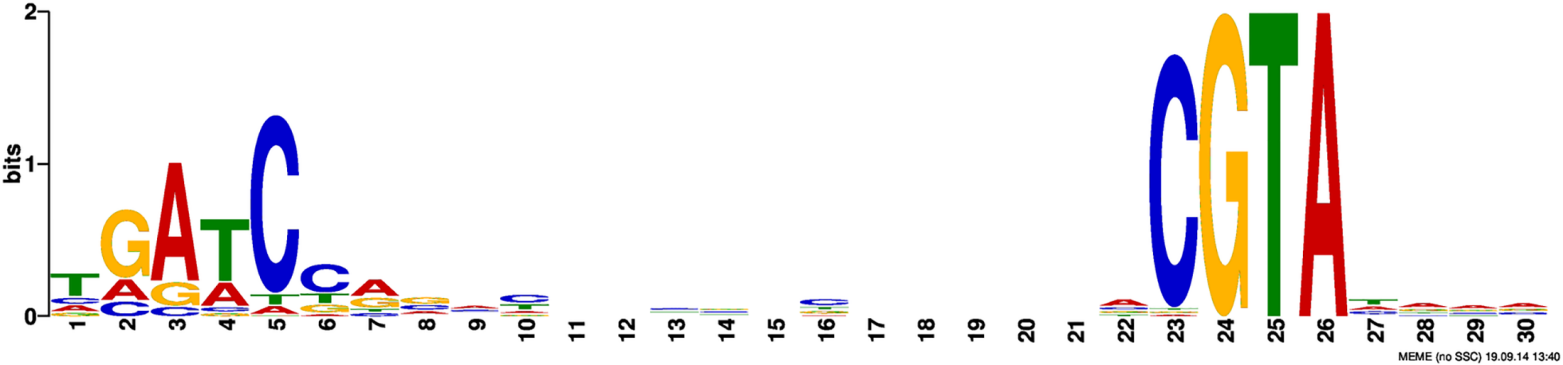
Promoter motif identified by MEME using the peaks identified by CSDeconv.

Only one significant motif was detected (Fig 1), and it was found within 87 of the 137 peaks. This motif closely resembles the RpoE_Rsp_-controlled promoter region identified in *Rhodobacter* [14] and *Caulobacter crescentus* [27]. A more in-depth comparison of the motifs appears in S2 Text. Of these 87 peaks, 54 were located within annotated coding regions and another 12 were arranged antisense to a coding region. The remaining 21 were located upstream of annotated open reading frames in locations typical for promoters. One peak is immediately upstream of the PSPTO_1043 gene itself, suggesting that, like many ECF sigma factors, this gene is autoregulated (S1(A) Fig).

A previous study [14] used computational methods to predict a core regulon for the RpoE_Rsp_-ChrR system across many bacteria based on the presence of an RpoE_Rsp_ promoter motif upstream from orthologous genes. The core regulon was predicted to contain six genes (in addition to *rpoE*_Rsp_ and *chrR*) and in *Rhodobacter sphaeroides* these are arranged in three loci. Orthologs for all six genes are present in the DC3000 genome (31-39% identity) (Table 2). Five are found in one locus (from PSPTO_1123—PSPTO_1115). We observed PSPTO_1043 binding upstream of PSPTO_1121 (*phrB*) within its neighbor PSPTO_1122 (S1(B) Fig). No PSPTO_1043 binding was observed upstream of the sixth ortholog (PSPTO_2515).

**Table 2:** Orthologs in DC3000 of the core RpoE_Rsp_-ChrR regulon [14].

*R. sphaeroides*	Gene name	*P. syringae* DC3000	% identity	Annotated Product in DC3000
RSP_1092	*rpoE*	PSPTO_1043	38%	RNA polymerase sigma factor RpoE_Rsp_
RSP_1093	*chrR*	PSPTO_1042	35%	transcriptional activator ChrR
RSP_1087		PSPTO_1119	35%	short chain dehydrogenase/reduc-tase family oxidoreductase
"		PSPTO_4065	35%	short-chain dehydrogenase/reduc-tase family oxidoreductase
"		PSPTO_1861	31%	short chain dehydrogenase
RSP_1088		PSPTO_2515	32%	lipoprotein
RSP_1090		PSPTO_1117	35%	hypothetical protein
RSP_1091		PSPTO_1118	39%	amine oxidase, flavin-containing protein
RSP_2143	*phrB*	PSPTO_1121 (*phrB*)	34%	deoxyribodipyrimidine photolyase
RSP_2144	*cfaS*	PSPTO_1116 (*cfa*)	39%	cyclopropane-fatty-acyl-phospholipid synthase

Amino acid sequences corresponding to several genes from *Rhodobacter sphaeroides* were aligned against the translated DC3000 genome using tblastn on the NCBI’s BLAST server [26]. For each *R. sphaeroides* gene, the gene from *P. syringae* DC3000 containing the highest scoring alignment is listed along with the percentage of identity between the two genes, along with the predicted function of the gene.

In the remainder of our experiments, we focused attention on 18 promoters that were located in regions typical for promoter regions, are predicted to control expression of genes homologous to members of the RpoE_Rsp_ regulon, and /or are predicted to control genes conserved in other *Pseudomonas syringae* strains. This set includes the promoter located upstream from *phrB* (within PSPTO_1122). The list of candidate promoters and the genes they potentially regulate can be found in Table 3.

**Table 3:** PSPTO_1043 promoters examined in this study.

Gene with putative PSPTO_1043 binding motif	Other genes in locus	Predicted promoter sequence^1^	Annotated gene product	CSDeconv score^2^	qRT-PCR fold change^3^	^1^O_2_ and tBOOH assays^4^	%*P. syringae* genomes with homolog and motif^5^
**PSPTO_0744**		cGATCgacacggctcatccattCGTAtgca	**acetyl-CoA acetyltransferase**	**22.09**	**11.42**	**N/T**	**57.85**
**PSPTO_1043**	**PSPTO_1042**	TGATCCactcttcatcccgctaCGTAacac	**RNA polymerase sigma factor RpoE_Rsp_, transcriptional activator ChrR**	**85.42**	**N/A**	+	**93.39**
**PSPTO_1121 (** ***phrB***)	**PSPTO_1120-PSPTO_1114**	aGATCCataacgccgagctgctCGTAcagg	**see** Table 2	**67.29**	**14.29**	+	**90.91**
PSPTO_1372 (*hopAA1-1*)		aGcaCtgcgctgttcaaacttcCGTAgaac	type III effector HopAA1-1	2.99	N/S	N/T	9.09
**PSPTO_1900**	**PSPTO_1901 (** ***bphO*** **), PSPTO_1902 (** ***bphP***)	TGATCCgcatctttacgaaacaCGTAcatc	**hypothetical protein, bacteriophytochrome heme oxygenase (BphO), bacteriophytochrome histidine kinase (BphP)**	**9.05**	12.85*	**0**	**N/D**
**PSPTO_2591**		TGATCCagtgtgcgcctgcctgCGTAtgtt	**diguanylate cyclase**	**86.94**	**21.42**	**N/T**	**82.64**
**PSPTO_2593**	**PSPTO_2592 (** ***saxG***)	TGAgCCaatattgactcaaagcCGTAcaaa	**multidrug resistance protein, AcrA/AcrE family; aliphatic isothiocyanate resistance protein SaxG, AcrB/AcrD/ AcrF family**	**28.19**	**1.38**	**N/T**	**61.98**
**PSPTO_2615**		TGATCCctgcctatacaacataCGTAtgtc	**GAF domain-containing protein**	**5.00**	**2.12**	**N/T**	**61.16**
PSPTO_2853		caaaCCataagcattctcaactCGTActaa	TonB-dependent receptor	12.45	N/S	N/T	1.65
**PSPTO_3893**		TGATCCaaagcatggctgctatCGTAagca	**glyoxalase**	**23.14**	**107.42**	**N/T**	**90.08**
**PSPTO_3907**		gaAcCaggcgtgcgtccaataaCGTAtaag	**hypothetical protein**	**19.28**	**3.44**	**N/T**	**73.55**
**PSPTO_4231 (** ***tctD***)	**PSPTO_4230**	gcATCCgggcgcctgttttacaCGTAcccg	**DNA-binding response regulator TctD; sensor histidine kinase TctE**	**4.56**	**7.15**	**N/T**	**86.78**
**PSPTO_4335**		TGATCtaggctgtgtttaccaaCGTActaa	**hypothetical protein**	**95.33**	**102.39**	**N/T**	**53.72**
**PSPTO_4530 (** ***katG***)		TaATCtgatgatcgctgtgcgaCGTAtctg	**catalase/peroxidase HPI**	**60.35**	**41.23**	+	**75.21**
PSPTO_4675		caAaCtaggtgatctcgatcttCGTAgaaa	Sir2 family transcriptional regulator	7.98	4.23	N/T	9.09
PSPTO_4702		aacTCtacccctaccgacttttCGTAcaaa	ISPssy, transposase	3.41	3.35	N/T	2.48
PSPTO_4723		gaAaCgacattgagtcttttttCGTAcaaa	hypothetical protein	4.26	4.19	N/T	3.31
**PSPTO_4843**		TGATCCacctgcccgcaagcaaCGTAgcgg	**esterase/lipase/ thioesterase family protein**	**20.92**	**10.13**	**N/T**	**84.30**

**Boldface** indicates loci induced by PSPTO_1043 overexpression.

^1^ Each locus’s predicted promoter sequence. Underlined uppercase letters are used to indicate which bases match the consensus sequence, TGATCCnnnnnnnnnnnnnnnnCGTAnnnn.

^2^ Score reported by CSDeconv for the peak upstream of the gene.

**Fig 2:**
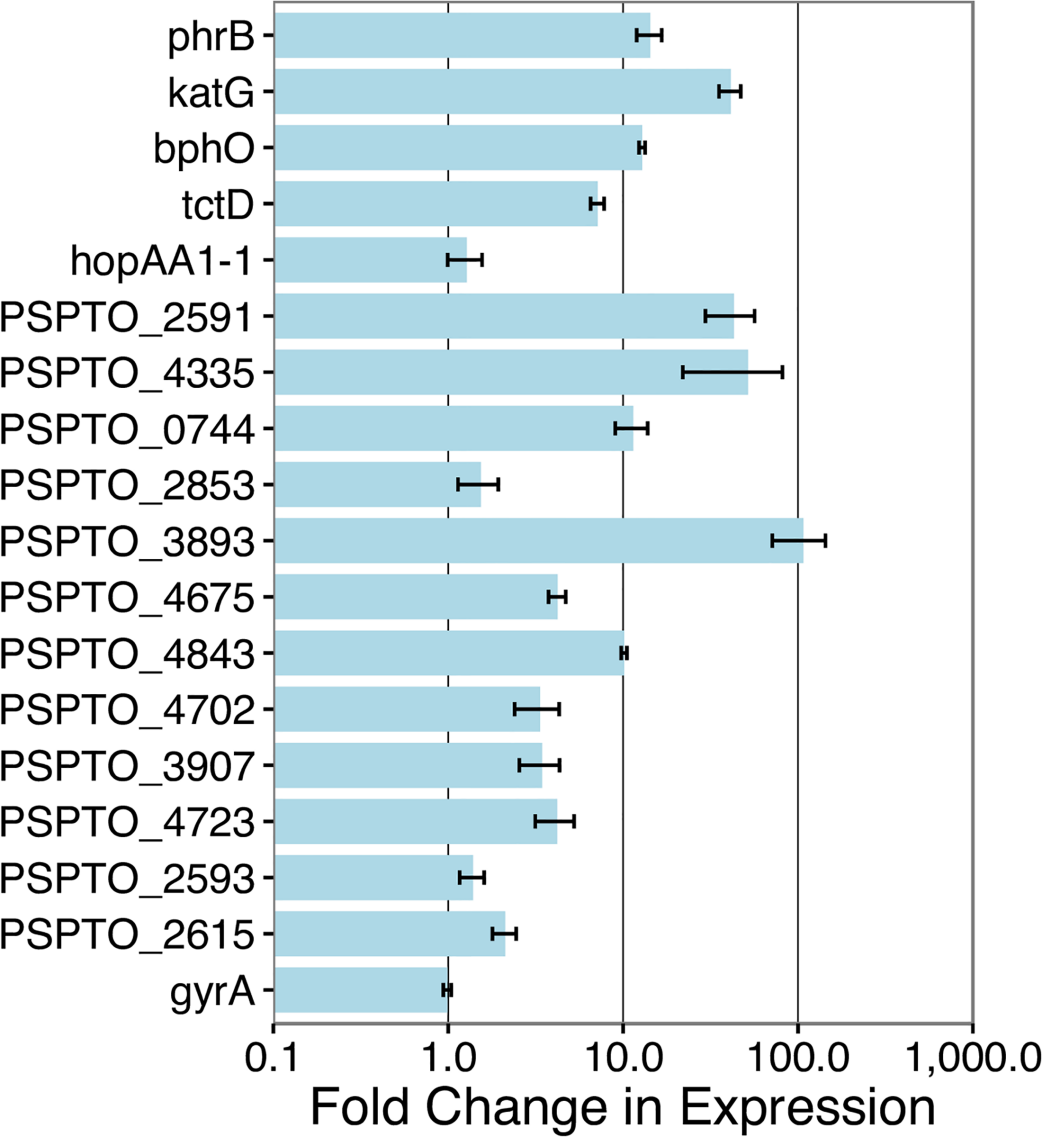
PSPTO_1043-dependent expression in DC3000. Seventeen putative PSPTO_1043-controlled genes and one housekeeping gene (*gyrA*) were evaluated and the fold change in expression between strains overexpressing PSPTO_1043 and those carrying the empty vector was calculated as described in the methods (normalized to the housekeeping gene *gap-1*).

^3^ qRT-PCR fold change (if statistically significant) after overexpression of PSPTO_1043 as shown in Fig 2. N/A = not applicable. PSPTO_1043 was overexpressed in the RT-PCR experiments. N/S = not significant.

^4^ Induction observed in the ^1^O_2_ and tBOOH *lux* assays shown in Figs 4 and 5. “+” = induced, “0” = not induced, “N/T” = not tested.

^5^ Percentage of *P. syringae* genomes that contain a homolog for the gene with an upstream PSPTO_1043 motif. N/D = no data, explanation in S1 Text

* PSPTO_1901 (*bphO*) tested.

### Expression of putative PSPTO_1043 controlled genes

The ChIP-Seq experiment detects PSPTO_1043 binding but does not demonstrate transcription of downstream regions. To examine gene expression directly, RNA was isolated from cells grown under the same conditions as used in the ChIP experiment. RNA levels were determined by qRT-PCR and normalized using expression from the PSPTO_1043-independent gene *gyrA*. We examined genes associated with 17 of the 18 identified promoters (all but PSPTO_1043) and found increased expression for 13 of them when PSPTO_1043 was overexpressed (Fig 2).

**Fig 3:**
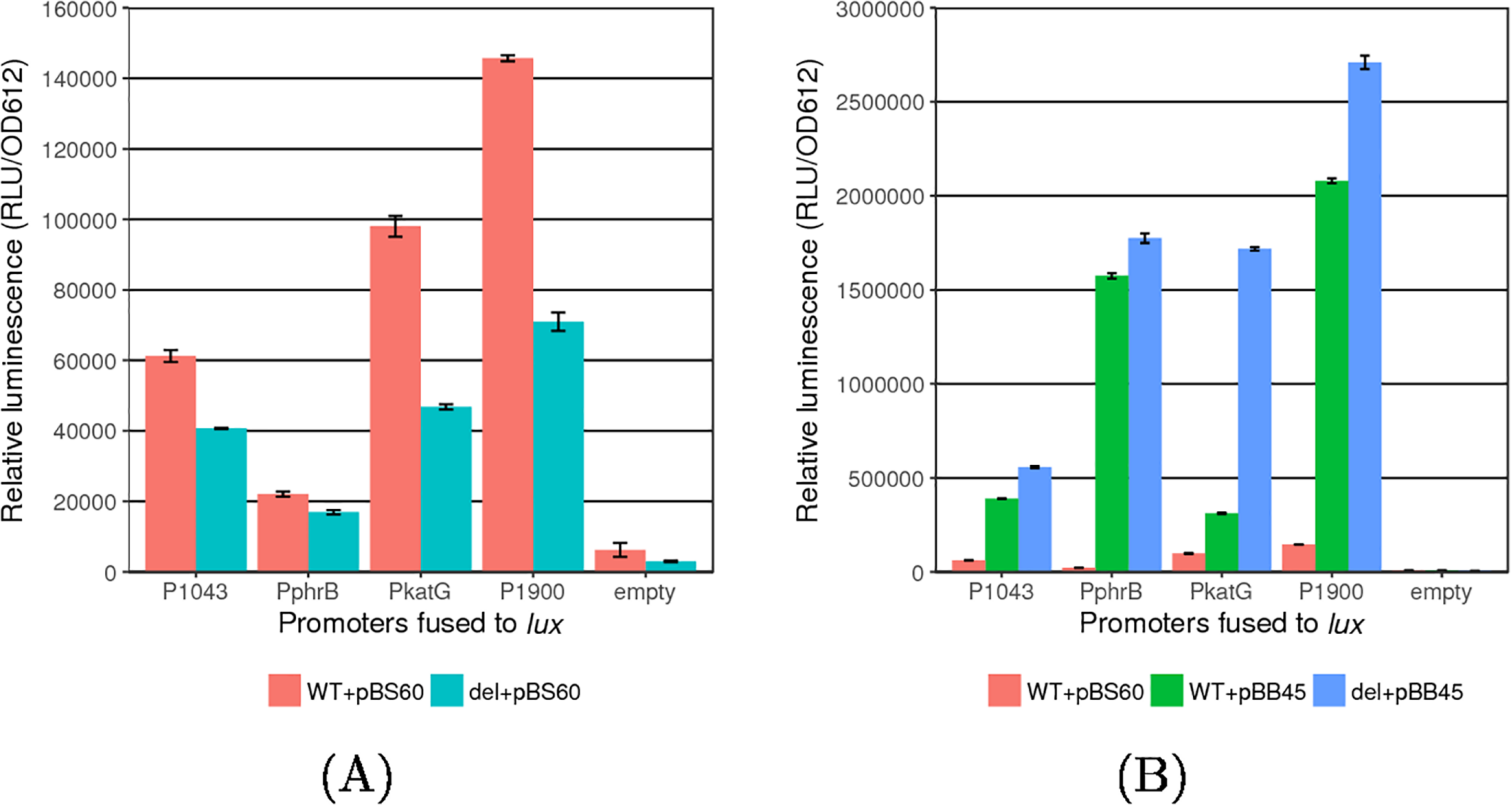
PSPTO_1043-dependent promoter activity. Candidate promoter-containing regions were fused to the *lux* reporter gene in a plasmid. Lux expression was monitored after 6hr in WT or ΔPSPTO_1043/1042 strains carrying either the plasmid overexpressing PSPTO_1043 (PnptII-1043) or an empty vector. (A) A slight decrease in expression was observed in strains lacking PSPTO_1043/1042 (green bars) compared to WT (red bars) when no PSPTO_1043 was overexpressed. (B) Overexpression of PSPTO_1043 in both WT (green bars) and ΔPSPTO_1043/1042 (blue bars) results in increased expression from all the tested promoter fusions compared to strains lacking the overexpression construct (red bars).

In order to study PSPTO_1043-dependent expression more closely, we cloned the putative promoters for four genes (*phrB*, PSPTO_1043, *katG* and PSPTO_1900) upstream from a *lux* reporter gene and tested whether overexpression of PSPTO_1043 affected *lux* expression (Fig 3). Expression from all four promoters was reduced in a PSPTO_1043/1042 double mutant compared to WT strains (two-tailed t-test, *p* < 0.05) (Fig 3A). Expression *in trans* of PSPTO_1043 was sufficient to complement the PSPTO_1043/1042 deletion. Addition of the PSPTO_1043 overexpression construct increased expression from all four promoters significantly (two-tailed t-test, *p* < 0.05) (Fig 3B) with the *phrB* promoter as the most responsive (100-fold change in the ΔPSPTO_1043/1042 strain and 70-fold change in the WT strain).

### PSPTO_1043 responds to the presence of ^1^O_2_ and tBOOH

**Fig 4:**
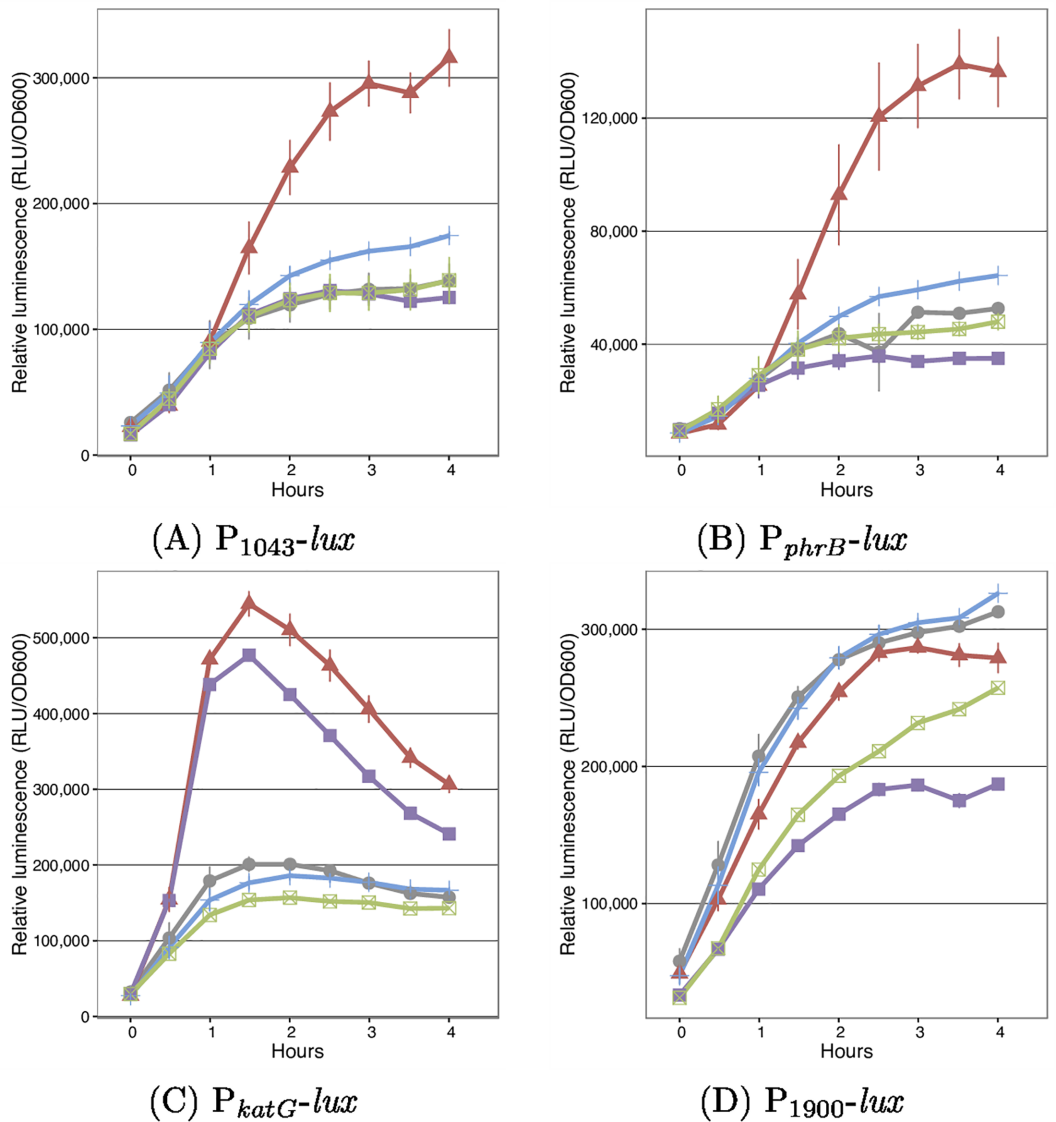
Expression from candidate PSPTO_1043 promoters in the presence of ^1^O_2_. WT (blue and red) or ΔPSPTO_1043/1042 (green or purple) strains carrying promoter-*lux* fusions were grown in the presence of 2.5*μ*M Rose Bengal under light (red and purple) or dark (blue and green) conditions. In the presence of light ^1^O_2_ is produced. As a control WT cells were grown in the presence of light, but without Rose Bengal (grey).

The assays described above detected an increase in expression from cloned promoters under conditions where the PSPTO_1043 sigma factor was artificially overexpressed. Because overexpression can generate artifacts, we attempted to identify conditions in which the sigma factor can be induced from its native context. Given the similarities between PSPTO_1043/1042 and RpoE_Rsp_-ChrR, and that RpoE_Rsp_-ChrR responds to ^1^O_2_, we used the *lux* promoter fusions to investigate whether DC3000 responds to ^1^O_2_. Expression from these promoters was monitored in WT and PSPTO_1043/1042 deletion strains following exposure to Rose Bengal in the absence (no ^1^O_2_ produced) or presence (^1^O_2_ produced) of light. Expression from the PSPTO_1043 and *phrB* promoters was increased in the presence of ^1^O_2_ and was dependent on PSPTO_1043 (Fig 4A and 4B). The *katG* promoter was also induced in the presence of ^1^O_2_ but showed less dependence on PSPTO_1043, suggesting that the cloned promoter region for this gene contains additional (PSPTO_1043 independent) promoters (Fig 4C). Because expression in these experiments depends on the presence of Rose Bengal, the response is not due to light exposure itself. No induction was observed from the PSPTO_1900 promoter (Fig 4D).

**Fig 5:**
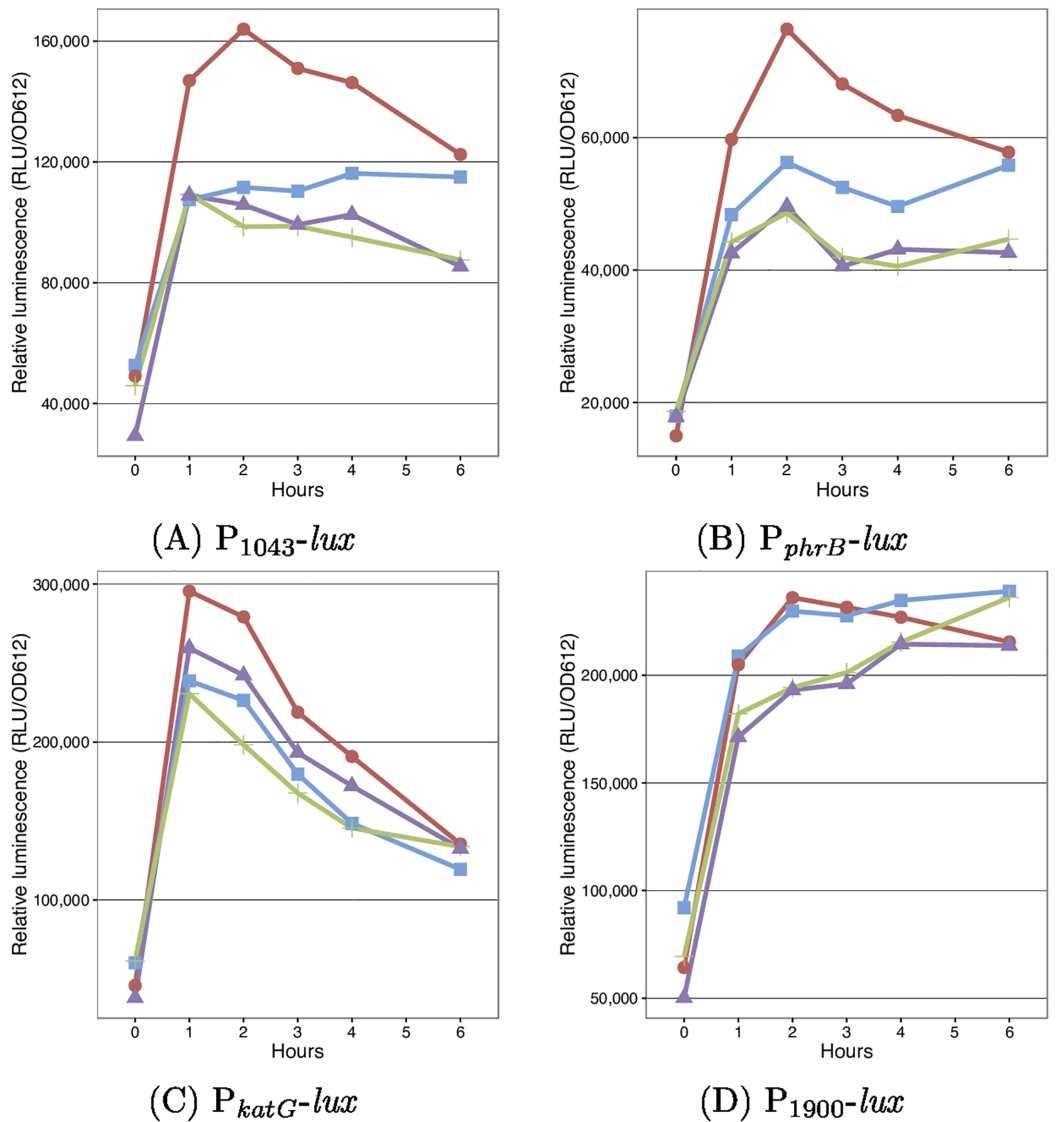
Expression from candidate PSPTO_1043 promoters in the presence of tBOOH. WT (blue and red) or ΔPSPTO_1043/1042 (green and purple) strains carrying promoter-*lux* fusions were grown in the presence (red and purple) or absence (blue and green) of 0.1mM tBOOH.

The RpoE_Rsp_-ChrR system in other bacteria has also been shown [27] to respond to other chemical such as tert-butyl hydroperoxide (tBOOH). The response of DC3000 to this compound was also investigated. The PSPTO_1043 and *phrB* promoters were induced in the presence of tBOOH (Fig 5A and 5B). Again, the *katG* promoter responded to tBOOH but was independent of PSPTO_1043 (Fig 5C). The PSPTO_1900 promoter was unresponsive (Fig 5D).

We also tested whether or not the presence of ^1^O_2_ and tBOOH or differential light exposure produced non-specific (PSPTO_1043-independent) changes in gene expression or altered the growth of the WT or ΔPSPTO_1043/1042 strains, and thus indirectly affect the expression of the regulon genes. No differences in bacterial growth or expression from the *gap-1* housekeeping gene promoter were detected (S3 Text).

### Identification of the PSPTO_1043 “core regulon” in *P. syringae* strains

A simple BLAST search against the NR database suggests that PSPTO_1043 and PSPTO_1042 are specific to *P. syringae* strains (Table 1). In order to study the conservation of the PSPTO_1043/1042 and its regulon in more detail, we searched for the PSPTO_1043 promoter motif upstream of orthologs of the members of PSPTO_1043/1042 regulon in other sequenced Pseudomonadales genomes. To do this, we first found all potential orthologs of the DC3000 regulon (based on protein sequence) then searched for the PSPTO_1043 promoter motif upstream of the coding sequence. The results of this analysis can be found in S2 Table and the full details of the analysis can be found in S1 Text.

These results suggest that PSPTO_1043 is part of a six-gene core regulon whose genes are conserved within *P. syringae* strains. These six genes do not appear to have homologs in other pseudomonads, such as *P. fluorescens* and *P. stutzeri*, suggesting that these genes and their regulation are adapted to provide functions necessary specifically for P. syringae’s lifestyle. The six genes (PSPTO_1043, *phrB*, diguanylate cyclase PSPTO_2591, putative glyoxalase PSPTO_3893, the *tctD* response regulator PSPTO_4231 and esterase/lipase/thioesterase family protein PSPTO_4843) are found in more than 80% of the *P. syringae* strains and closely related genomes (more than 100 out of 121) (Table 3). Six additional genes are conserved in the closely related *tomato*, *thea*, *avellanae* and *actinidiae* pathovars (Table 3). These genes encode an acetyl-CoA acetyltransferase (PSPTO_0744), multidrug resistance protein (PSPTO_2593), GAF domain protein (PSPTO_2615), catalase (*katG*, PSPTO_4530) and two hypothetical proteins (PSPTO_3907 and PSPTO_4335).

Conservation of the other genes in the putative DC3000 regulon appear more variable (S2 Table). Homologs of PSPTO_2853 can be found in a number of *P. aeruginosa* and *P. putida* strains but do not appear to be preceded by a PSPTO_1043 promoter motif. Homologs of PSPTO_4702 without PSPTO_1043 promoter motifs can be found in some *P. aeruginosa* strains. Homologs of PSPTO_4723 are found in tomato pathovars, but do not appear to be preceded by a PSPTO_1043 promoter motif. The absence of 1043 promoter motif in species lacking 1043 homologs is consistent with the acquisition of this system in syringae after they diverged from the other pseudomonads. Homologs of *hopAA1-1* (PSPTO_1372) and PSPTO_4675 are found in various *P. syringae* strains, but they do not have an associated PSPTO_1043 promoter motif except in the other tomato pathovars. Finally, although several *P. fluorescens* strains have homologs of *phrB* that are preceded by a putative PSPTO_1043 binding site, they do not appear to encode a PSPTO_1043 homolog. These promoter motifs may be recognized by a different sigma factor or have been conserved for other reasons.

## Discussion

In this report, we describe the ECF sigma factor, PSPTO_1043, which is predicted to be co-transcribed with PSPTO_1042, a likely anti-sigma factor. PSPTO_1043 and PSPTO_1042 are homologous to the RpoE_Rsp_-ChrR genes in *Rhodobacter sphaeroides* [31] and *Azospirillum brasilense* [32]. The RpoE_Rsp_-ChrR system [14, 28] responses to the presence of ^1^O_2_, which is produced during photosynthesis, and may play an important role in protecting these photosynthetic bacteria from its harmful effects. A homologous system in *Caulobacter crescentus*, a free-living bacterium found in nutrient-poor aquatic environments, also responds to the presence of ^1^O_2_ and also cadmium [27].

^1^O_2_ is a toxic reactive oxygen species (ROS) that can be produced by photo-activation of oxygen by energy transfer from excited photosynthetic pigments during photosynthesis or by certain biochemical reactions involving peroxides and epoxides [33]. ^1^O_2_ is an oxidizing agent that can react with a variety of compounds in biological systems including unsaturated lipids, the side chains of the amino acids, Trp, Tyr, His, Met and Cys, cellular thiols such as glutathione, DNA bases and secondary metabolites such as terpenes [34]. Therefore mechanisms to quench ^1^O_2_ and protect against its effects are found in many organisms. In addition, plants use ^1^O_2_ to defend themselves from pathogens [34, 35]. Since *P. syringae* is not a photosynthetic bacterium, the presence of this sigma factor may be related to its specific lifestyle as a plant pathogen.

The DC3000 ΔPSPTO_1043/1042 strain is slightly inhibited in growth in *Arabidopsis* seedlings at early stages of infection, but the difference was no longer apparent 4 days post-inoculation (S4 Text). Despite the effect on bacterial growth at early stages of infection, no difference in symptoms was observed when tomato plants were infected with WT or the PSPTO_1043/1042 double mutant strains. We speculate that PSPTO_1043/1042 is important at early stages of infection or colonization and less so at later stages.

Thakur et al. [36] have studied Psyr_0892, the PSPTO_1043 ortholog in *Pseudomonas syringae* pv. *syringae* B728a. They have found that ΔPsyr_0892 mutants appear to be equality sensitive as the WT strain to the presence of H_2_O_2_ on agar plates. However, they did not directly measure changes in the expression of Psyr_0892 to presence of H_2_O_2_ nor did they perform their assaying using either ^1^O_2_ or tBOOH.

In previous work [14], homologs of the *rpoE*_Rsp_-*chrR* gene pair in *Rhodobacter sphaeroides* from 73 bacterial genomes were analyzed. The authors found that a phylogenetic tree based on the amino acid sequences of *rpoE*_Rsp_ and *chrR* generally mirrored a tree based upon the sequences of *ruvB*, *rpoD* and *gyrB*, except for *P. syringae* and *Oceanospirillum* spp. The same study proposed a core regulon of eight genes (including *rpoE*_Rsp_ and *chrR*) that is conserved in 45 of 73 species analyzed. The authors of this study speculate the the genes in this regulon provide functions to both prevent and repair damage from oxidative stress. Members of this regulon encode *phrB*, a deoxyribodipyrimidine photolyase, *cfaS*, a cyclopropane fatty acyl-phospholipid synthetase, RSP1087, a short-chain dehydrogenase/reductase, and RSP1091, a flavin-containing oxidoreductases.

Homologs for all eight genes are present in DC3000 (with about 30-40% identity) and are found in three loci (Table 2). The first locus is composed of PSPTO_1043 and PSPTO_1042. The second locus is composed of PSPTO_1121(*phrB*)-PSPTO_1116(*cfa*). In *P. aeruginosa* and *P. syringae*, *phrB* has been shown to be involved with RNA repair from UV-B damage in photo-reactivating conditions [37]. *cfa* is annotated as a cyclopropane-fatty-acyl-phospholipid synthase, which is part of the fatty acid biosynthesis pathway. The other genes in this locus are less well characterized, and their putative functions are shown in Table 2. The third locus consists of a single gene, PSPTO_2515, of unknown function. A PSPTO_1043 promoter motif was observed upstream of the first and second, but not the third locus.

We used a ChIP-Seq approach to identify putative PSPTO_1043 binding sites in cells where the sigma factor was constitutively expressed. This approach yielded 137 enriched regions or peaks. Using these regions, we identified a putative PSPTO_1043 promoter motif (Fig 1) associated with 87 of the 137 peaks. The motif closely resembles the putative RpoE_Rsp_ promoter identified in *Rhodobacter* and *Caulobacter* (S2 Text). Many of these sites were located in non-canonical positions, such as within annotated coding regions or in orientations that would support antisense transcription. These results must be interpreted cautiously because ChIP-based experiments are susceptible to false positive results and by themselves do not demonstrate promoter functionality [38]. In addition, because our experiments relied on the overexpression of PSPTO_1043, binding may have occurred at lower affinity sites that are not biologically relevant.

Table 3 summarizes the results of both our molecular and computational experiments. Of the three transcriptional units identified in DC3000 as containing homologs of the previously reported RpoE_Rsp_-ChrR core regulon, we observed PSPTO_1043 binding upstream of PSPTO_1043 and PSPTO_1121 (*phrB*), but failed to detect binding upstream of PSPTO_2515.

Additionally, our results strongly support the addition of at least four more *P. syringae* specific transcription units to the PSPTO_1043 regulon. PSPTO_2591 encodes a predicted diguanylate cyclase and is likely to generate cyclic-di-GMP, an intracellular messenger molecule [39]. In concert with phosophodiesterases, this protein regulates the level of c-di-GMP present in the bacterial cell and controls functions such as motility, biofilm formation, and virulence gene expression [39]. Further experiments will be needed to determine whether or not PSPTO_1043/1042 regulates any of these functions. PSPTO_3893 encodes a putative glyoxylase and PSPTO_4843 encodes a protein belonging to the esterase-lipase superfamily. PSPTO_4231 and PSPTO_4230 encode a two-component system with homology to the TctDE tricarboxylic acid transporter two-component regulatory system. In *Xanthomonas campestris* pv. *vesicatoria*, the causal agent of bacterial spot disease of tomato and pepper, TctDE was found to influence the expression of *citH* (encoding a citrate transporter), which is important for growth of this pathogen in tomato plants [40].

We also identified an additional seven promoters in DC3000 that are predicted to regulate genes with varying homology across *P. syringae* strains. PSPTO_1900 does not appear to be induced in the presence of ^1^O_2_ or tBOOH in a manner similar to PSPTO_1043, *phrB* or *katG*. It is possible that this behavior is consistent with the relatively small ChIP-Seq enrichment signal associated with PSPTO_1900. Nevertheless, expression of this gene is up-regulated when PSPTO_1043 is overexpressed (Fig 2), and its promoter is induced in the *lux* assay (Fig 3), so we have included it in the expanded regulon.

PSPTO_4530 (*katG*), which encodes the major housekeeping catalase, KatG, is involved in detoxifying exogenous H_2_O_2_ [41]. Together with KatB, KatG was found to be a major catalase involved in virulence of DC3000 [41]. We show that although expression of *katG* is affected by PSPTO_1043, there is still significant expression in a PSPTO_1043/1042 double mutant suggesting that at least one other promoter acts to control expression of this gene. In *P. aeruginosa* [42] and *P. syringae* [43], OxyR has been shown to induce *katG*, but the OxyR binding consensus sequence was not found upstream of *katG*. Taken together, this might explain why Thakur et al. [36] did not observe a difference between ΔPsyr_0892 and WT B728a in the the presence of H_2_O_2_. Further experiments are needed to determine the relationship between PSPTO_1043, *oxyR* and *katG*.

An additional five genes regulated by PSPTO_1043 are conserved in the closely related *tomato*, *thea*, *avellanae* and *actinidiae* pathovars. These genes encode an acetyl-CoA acetyltransferase (PSPTO_0744), multidrug resistance protein (PSPTO_2593), GAF domain protein (PSPTO_2615) and two hypothetical proteins (PSPTO_3907 and PSPTO_4335). In the Conserved Domain Database [44], PSPTO_3907 is annotated with a WbqC-like protein family (pfam08889), which may be involved in O-antigen production. PSPTO_4335 is annotated with a PilZ domain (cl01260), which is a c-di-GMP binding domain that may be involved with flagellar torque generation.

While we have demonstrated that PSPTO_1043/1042 gene expression is induced in the presence of ^1^O_2_ and tBOOH, additional work is needed to determine whether PSPTO_1043/1042 is directly induced by either. It is possible that the presence of these reactive oxygen species produce reactions whose products are the direct inducers of PSPTO_1043/1042. However, taken together our results show PSPTO_1043/1042 responds to the presence of these chemicals and that PSPTO_1043 controls transcription of genes whose homologs are found in the RpoE_Rsp_-ChrR regulons and that have been shown to be involved in oxidative stress response of other bacteria. In addition, the sigma factor controls expression of a *P. syringae* specific set of genes whose function in DC3000 is largely unstudied. It is possible that these genes play a role in survival of the bacteria within the plant (e.g., citrate utilization, H_2_O_2_ defense and modulation of c-diGMP levels that may alter motility or biofilm formation) and further studies will be required to test these hypotheses.

## Supporting information

S1 Fig Examples of ChIP-Seq peaks upstream of selected targets. (A) Peak upstream of the PSPTO_1043/1042 locus. (B) Peak upstream of *phrB* and other homologs of the RpoE_Rsp_-ChrR core regulon. The genomic profiles shown can be found in S2 Dataset.
(TIFF)

S1 Table Strains, plasmids, and primers used in this study. (DOCX)

S2 Table Orthologs of the PSPTO_1043 regulon in the sequenced Pseudomonadales. Reciprocal Best BLAST Hits (RBBH’s) were computed between DC3000 and 1028 genomes from the order Pseudomonadales and then used to identify orthologs of the genes in the PSPTO_1043 regulon. Once families of orthologous genes were identified, the regions upstream of each gene were extracted and scanned for the PSPTO_1043 motif. This table includes the results for each of the analyzed genomes, grouped according to their taxonomy. For each genome, the orthologs found are reported together with FIMO score of any PSPTO_1043 motif found.
(XLSX)

S1 Text A description of the method used to compute S2 Table. (DOCX)

S2 Text A detailed discussion of the claim that the PSPTO_1043 binding sequence closely resembles the RpoE_Rsp_ binding sequence identified in *Rhodobacter* and *Caulobacter crescentus*. (DOCX)

S3 Text Assays to measure the effect of ^1^O_2_ and tBOOH on wildtype and ΔPSPTO_1043/1042 *Pseudomonas syringae* pv *tomato* DC3000. (DOCX)

S4 Text Assays to measure growth of wildtype and ΔPSPTO_1043/1042 *Pseudomonas syringae* pv *tomato* DC3000 in *Arabidopsis* seedlings. (PDF)

S1 Dataset The “sinister” profile of the PSPTO_1043 ChIP-Seq reads that align to the DC3000 chromosome sequence. Sinister profiles and file format is described in [17].
(ZIP)

S2 Dataset The “naive” profile of the PSPTO_1043 ChIP-Seq reads that align to the DC3000 chromosome sequence. Naive profiles and file format is described in [17].
(ZIP)

S3 Dataset The 137 regions of enrichment identified by CSDeconv in GFF format (https://www.sanger.ac.uk/resources/software/gff/spec.html). (GFF)
