# Supplementary figures and images for: The ECF sigma factor, PSPTO_1043, in Pseudomonas syringae pv. tomato DC3000 is induced by oxidative stress and regulates genes involved in oxidative stress response
Source: PLoS One. 2017 Jul 12;12(7):e0180340. doi: 10.1371/journal.pone.0180340 (PMC5507510; doi:10.1371/journal.pone.0180340)

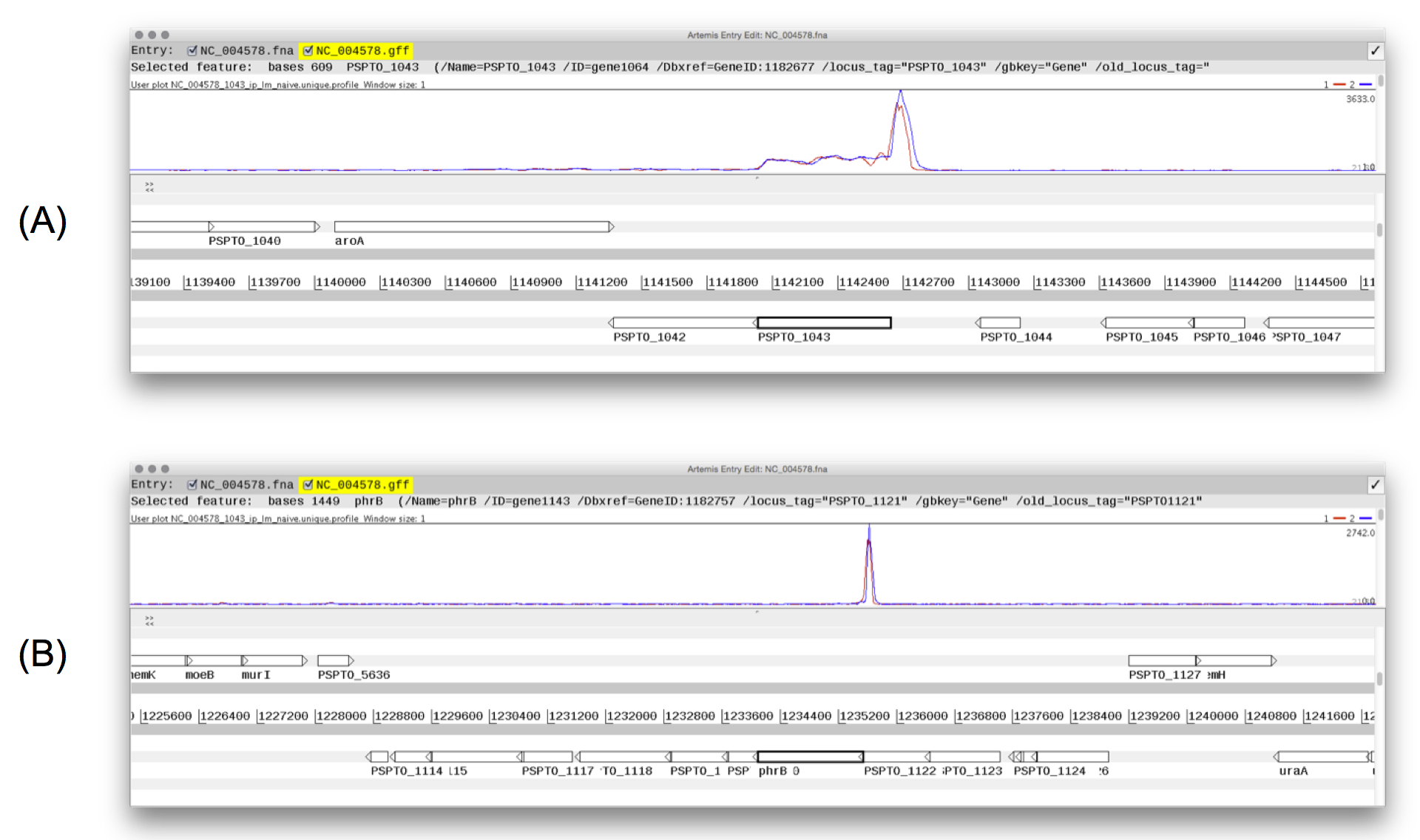

Supplement: S1 Fig — (A) Peak upstream of the PSPTO_1043/1042 locus. (B) Peak upstream of phrB and other homologs of the RpoERsp-ChrR core regulon. The genomic profiles shown can be found in S2 Dataset. (TIFF) [file pone.0180340.s001.tiff]
