# Supplementary material for: The ECF sigma factor, PSPTO_1043, in Pseudomonas syringae pv. tomato DC3000 is induced by oxidative stress and regulates genes involved in oxidative stress response
Source: PLoS One. 2017 Jul 12;12(7):e0180340. doi: 10.1371/journal.pone.0180340 (PMC5507510; doi:10.1371/journal.pone.0180340)
Supplement: S1 Table — (DOCX) [file pone.0180340.s002.docx]

Strains, plasmids, and primers used in this study.

This table contains the Strains, plasmids, and primers used in the paper, “The ECF sigma factor, PSPTO_1043, in *Pseudomonas* *syringae* pv. *tomato* DC3000 is induced by oxidative stress and regulates genes involved in oxidative stress and virulence” by Butcher, et al.

## Strains

| Name | Description | Reference |
| --- | --- | --- |
| DC3000 | *P.* *syringae* pv. *tomato* DC3000 wild-type strain DC3000, rif^R^ | [1] |
| BBPS12 | DC3000 (pBS60) | This study |
| BBPS21 | DC3000 (pBB45) | This study |
| BBPS32 | DC3000 ΔPSPTO_1043/1042 | This study |
| BBPS37 | DC3000 (pBB60) | This study |
| BBPS38 | DC3000 (pBB61) | This study |
| BBPS39 | DC3000 (pBB62) | This study |
| BBPS40 | DC3000 (pBB63) | This study |
| BBPS41 | BBPS32 (pBB60) | This study |
| BBPS42 | BBPS32 (pBB61) | This study |
| BBPS43 | BBPS32 (pBB62) | This study |
| BBPS44 | BBPS32 (pBB63) | This study |
| BBPS45 | BBPS32 (pBS44) | This study |
| BBPS46 | BBPS32 (pBS60) | This study |
| BBPS47 | BBPS32 (pBB45) | This study |
| BBPS55 | DC3000 (pBS163) | This study |
| BBPS65 | BBPS12 (pBB60) | This study |
| BBPS66 | BBPS12 (pBB61) | This study |
| BBPS67 | BBPS12 (pBB62) | This study |
| BBPS68 | BBPS12 (pBB63) | This study |
| BBPS70 | BBPS21 (pBB60) | This study |
| BBPS71 | BBPS21 (pBB61) | This study |
| BBPS72 | BBPS21 (pBB62) | This study |
| BBPS73 | BBPS21 (pBB63) | This study |
| BBPS74 | BBPS21 (pBS44) | This study |
| BBPS75 | BBPS55 (pBB60) | This study |
| BBPS76 | BBPS55 (pBB61) | This study |
| BBPS77 | BBPS55 (pBB62) | This study |
| BBPS78 | BBPS55 (pBB63) | This study |
| BBPS79 | BBPS55 (pBS44) | This study |

## Plasmids

| Name | Description | Reference |
| --- | --- | --- |
| pENTR/D | Topo cloning Gateway entry vector | Invitrogen |
| pENTR/SD/D | Topo cloning Gateway entry vector | Invitrogen |
| pBS46 |  |  |
| pBS60 |  |  |
| pBS58 | lux reporter construct | [2] |
| pBS44 |  |  |
| pBS5 | pENTR/SD/D-PSPTO_1043(Kan^R^) | This study |
| pBS163 | PnptII-PSPTO_1043 (Gent^R^) | This study |
| pBB36 | pENTR/SD/D-PSPTO_1043-FLAG (Kan^R^) | This study |
| pBB45 | PnptII-PSPTO_1043-FLAG (Gent^R^) | This study |
| pBB56 | pENTR/D-promoter PSPTO_1043 (Kan^R^) | This study |
| pBB57 | pENTR/D-promoter *phrB* (Kan^R^) | This study |
| pBB58 | pENTR/D-promoter *katG* (Kan^R^) | This study |
| pBB59 | pENTR/D-promoter PSPTO_1900 (Kan^R^) | This study |
| pBB60 | pBS58-promoter PSPTO_1043-*lux* (Kan^R^, Tet^R^) | This study |
| pBB61 | pBS58-promoter *phrB*-*lux* (Kan^R^, Tet^R^) | This study |
| pBB62 | pBS58-promoter *katG*-*lux* (Kan^R^, Tet^R^) | This study |
| pBB63 | pBS58-promoter PSPTO_1900-*lux* (Kan^R^, Tet^R^) | This study |
| pZB30 | pKB18mobsacB/PSPTO_1042-1043 (Kan^R^) | This study |

## Primers used in construction of the overexpression vector

| Name | Sequence | Description |
| --- | --- | --- |
| oSWC01724 | CACCATGCGCATTACTGCCAGTCTCA | PSPTO_1043 fwd |
| oSWC01725 | TCACTTGTCATCGTCGTCCTTGTAGTCTGACTCCTCGATCCGCGA | PSPTO_1043-FLAG rev |
| oSWC07 | CACCATGCGCATTACTGCCAGTCTCAGGAC |  |
| oSWC08 | TCATGACTCCTCGATCCGCGAGCGC |  |

## Primers used in construction of *lux* fusion vectors

| Name | Sequence | Description |
| --- | --- | --- |
| oSWC02620 | CACCCCATACAAACGATGACCAGCAGTA | Promoter PSPTO_1043 fwd |
| oSWC02621 | GGACAAGTGACAGAAGGTCCTGAGA | promoter PSPTO_1043 rev |
| oSWC02622 | CACCCGTTGCTGCTGGTCAATCAGT | promoter *phrB* fwd |
| oSWC02623 | CGTGAACGCGCAGGTCACTA | promoter *phrB* rev |
| oSWC02624 | CACCGCTACGCTCATGACAACGCTT | promoter *katG* fwd |
| oSWC02625 | GGGCATTTCGATTCAGTTGACAT | promoter *katG* rev |
| oSWC02626 | CACCCGGACATGTTTTCAGGCAAA | promoter PSPTO_1900 fwd |
| oSWC02627 | GCGACGAAACACCGTAGTGCAT | promoter PSPTO_1900 rev |

## Primers used in construction of the PSPTO_1043/1042 mutant

| Name | Sequence | Description |
| --- | --- | --- |
| oSWC02022 | AAGAGTGACGATACCCGTGTG |  |
| oSWC02023 | CACATGCCCGGGACTGCGACTGATGGGCGTG |  |
| oSWC02024 | ATGCAGCCGTTTTTCGGGATTTG |  |
| oSWC02025 | ATCCCGAAAAACGGCTGCATGAGACTGGCAGTAATGCGC |  |
| oSWC02026 | AACGCCTCTTGGTTTTGAGCC |  |
| oSWC02027 | CACATGCCCGGGATCCGGGGTTGAAGCTGTTG |  |
| oSWC02108 | ACCACGCTACGGATTTCCAC |  |
| oSWC02109 | TGTCGATGATCTGGCTGG |  |
| oSWC02110 | AGGTCAGGGAGACCATTATG |  |
| oSWC02111 | TTGGTCAATCCTGGCGAC |  |

## Primers used for RT-PCR

| Name | Sequence | Description |
| --- | --- | --- |
| oBGB00102 | AGGGTCAAGGCACTGTTG | tctD sense |
| oBGB00103 | GCGTGTCCAGATCGTAGG | tctD antisense |
| oBGB00104 | GCTGACTTTCCTTGGCTTTG | citM sense |
| oBGB00105 | CCGAACAGGGCGAAGATG | citM antisense |
| oBGB00106 | AACAACCGCCTGTGACTG | hopAA1-1 sense |
| oBGB00107 | CGGCAATCGCATTTATCTCG | hopAA1-1 antisense |
| oBGB00108 | GTCATGGCTGATCTTCGTAGTG | PSPTO_2591 sense |
| oBGB00109 | CGTGCTGGTCTGGATGTTAT | PSPTO_2591 antisense |
| oBGB00110 | GCAGGACTATCTGCAGGTTT | PSPTO_4335 sense |
| oBGB00111 | CTGATAAGCATCAGTCCTTCCC | PSPTO_4335 antisense |
| oBGB00116 | CGCCAATGCCAGCAATAAG | PSPTO_0744 FWD Set 2 |
| oBGB00117 | GAAGTCTCTGGAGTGCTTGAG | PSPTO_0744 REV Set 2 |
| oBGB00118 | GACGCCTGCAAGGATTTCTA | PSPTO_3893 FWD Set 2 |
| oBGB00119 | TTCTGATTGCCAAAGCGAAAC | PSPTO_3893 REV Set 2 |
| oBGB00120 | GCCTTATCCACGCAACAAAC | PSPTO_4843 FWD Set 2 |
| oBGB00121 | CTTCTCCAACTGCCTCCATAG | PSPTO_4843 REV Set 2 |
| oBGB00122 | TCAAGGACGCAACCCTTATC | PSPTO_3907 FWD Set 2 |
| oBGB00123 | TGCAAGGTTTCCCGAAAGTA | PSPTO_3907 REV Set 2 |
| oBGB00124 | CGGTCAGAGCGCCTATTTC | PSPTO_2593 FWD Set 2 |
| oBGB00125 | GGTCGAGTTGCTGAATGGT | PSPTO_2593 REV Set 2 |
| oBGB00126 | CATGCGCTTTGCGAGTATTG | PSPTO_2615 FWD Set 1 |
| oBGB00127 | CGAAGACTTCACCTTCGTCTATC | PSPTO_2615 REV Set 1 |
| oBGB00128 | GGTTGTATCTCTCGCGCTTAAA | PSPTO_2853 FWD Set 1 |
| oBGB00129 | CAGCCAGCCTGGACAATAAA | PSPTO_2853 REV Set 1 |
| oBGB00130 | TCTGACGTACCTAGACCAGATG | PSPTO_4675 FWD Set 5 |
| oBGB00131 | CAAACCAATGCTGGGCTTTC | PSPTO_4675 REV Set 5 |
| oBGB00132 | TCTGACGTACCTAGACCAGATG | PSPTO_4702 FWD Set 5 |
| oBGB00133 | CAAACCAATGCTGGGCTTTC | PSPTO_4702 REV Set 5 |
| oBGB00134 | TCTGACGTACCTAGACCAGATG | PSPTO_4723 FWD Set 5 |
| oBGB00135 | CAAACCAATGCTGGGCTTTC | PSPTO_4723 REV Set 5 |
| oSWC06425 | ACAACTTGAGCGTCTGGAG | 1043 sense |
| oSWC06426 | AGGTTCTGAATGGCACTATTAAG | 1043 antisense |
| oSWC06427 | AAGAATACGGCATCAACGAAAC | phrB sense |
| oSWC06428 | CAAGGTGGCTGGTGAAGTG | phrB antisense |
| oSWC06429 | GGCGTTACAAGGTATCGG | katG sense |
| oSWC06430 | CAGCAGCACAGTCATCTC | katG antisense |
| oSWC06431 | TCCGAACATGACATCAGACAAC | bphO sense |
| oSWC06432 | GCCTTCAAGCACATACATTACG | bphO antisense |
| oBGB00136 | CTCGAGAAGGTGAAACTGTAGAC | Easygene 1 FWD Set 1 |
| oBGB00137 | TCGAACAGCGATCGTAAGTG | Easygene 1 REV Set 1 |
| oBGB00138 | CGTTCTATATCCGAGGCCAATG | hop01-2 up FWD Set 4 |
| oBGB00139 | GTCTGACACACCTATGAAGCAG | hop01-2 up REV Set 4 |
| oBGB00140 | CGCTTCATCACGATAGGTCTT | hop01-2 down FWD Set 2 |
| oBGB00141 | CAAGTGTCTAGCCAGCTTCATA | hop01-2 down REV Set 2 |
| oBGB00142 | CCGGCAAGTAATGCTCTTGA | PSPTO_0412 down FWD Set 2 |
| oBGB00143 | GCCATCTGGATCGCAACAT | PSPTO_0412 down REV Set 2 |
| oBGB00144 | AGCCACCGATGTAGCTGTA | PSPTO_0412 up FWD Set 4 |
| oBGB00145 | AGAAGACGGGCTGGTTCTAT | PSPTO_0412 up REV Set 4 |
| oBGB00146 | CTGTCTGGCGTATTGCATCTA | PSPTO_4519 FWD Set 3 |
| oBGB00147 | AGGTGTCCTGCATCCAAAG | PSPTO_4519 REV Set 3 |
| oBGB00148 | CGGTCATATAGCTGCGTAGT | PSPTO_1084 FWD Set 3 |
| oBGB00149 | GAATCACGGCCTACCTGAA | PSPTO_1084 REV Set 3 |
| oBGB00150 | GACCGGCATTGTTTGTGTTT | PSPTO_1083-1084 int FWD Set 5 |
| oBGB00151 | ATCAAGACCTTTACGGCTGAG | PSPTO_1083-1084 int REV Set 5 |
| oBGB00152 | CAGGGCTTATGGTCGGTATT | PSPTO_3851 FWD Set 1 |
| oBGB00153 | ATCTGGCGTGAGCGATTT | PSPTO_3851 REV Set 1 |
| oBGB00154 | GGAGTTCTTCACGGCCAATA | PSPTO_1084 up FWD |
| oBGB00155 | GGACACCTACAACCTGACAC | PSPTO_1084 up REV |
| oBGB00156 | GCTGATAGCATTGATGGTGTTT | PSPTO_1084 mid FWD |
| oBGB00157 | GCCGCTGGTTCTGGATATAG | PSPTO_1084 mid REV |

## References

1. Buell C, Joardar V, Lindeberg M, Selengut J, Paulsen I, Gwinn M, et al. The complete genome sequence of the *Arabidopsis* and tomato pathogen *Pseudomonas syringae* pv. *tomato* DC3000. Proceedings of the National Academy of Sciences. National Acad Sciences; 2003;100: 10181. doi:[10.1073/pnas.1731982100](https://doi.org/10.1073/pnas.1731982100)

2. Swingle B, Thete D, Moll M, Myers CR, Schneider DJ, Cartinhour S. Characterization of the PvdS-regulated promoter motif in *Pseudomonas syringae* pv. *tomato* DC3000 reveals regulon members and insights regarding PvdS function in other pseudomonads. Mol Microbiol. 2008;68: 871–89. doi:[10.1111/j.1365-2958.2008.06209.x](https://doi.org/10.1111/j.1365-2958.2008.06209.x)
