# Supplementary material for: The ECF sigma factor, PSPTO_1043, in Pseudomonas syringae pv. tomato DC3000 is induced by oxidative stress and regulates genes involved in oxidative stress response
Source: PLoS One. 2017 Jul 12;12(7):e0180340. doi: 10.1371/journal.pone.0180340 (PMC5507510; doi:10.1371/journal.pone.0180340)
Supplement: S1 Text — (DOCX) [file pone.0180340.s004.docx]

Method for computing Orthologs of the 1043 regulon.

Paul Stodghill

## Introduction

This document describes the method used to generate S2 Table from the paper, “The ECF sigma factor, PSPTO_1043, in *Pseudomonas* *syringae* pv. *tomato* DC3000 is induced by oxidative stress and regulates genes involved in oxidative stress and virulence” by Butcher, et al.

## Downloading the genomes

On March 3, 2014, the closed and draft Pseudomonadales genomes were downloaded from <rsync://sync.ncbi.nih.gov/genbank/genomes>. In particular, the following rsync path name filters were used,

## Collections
Bacteria/
Bacteria_DRAFT/
## Order Pseudomonadales
### Family Moraxellaceae
Acinetobacter_*/
Alkanindiges_*/
Branhamella_*/
Enhydrobacter_*/
Moraxella_*/
Paraperlucidibaca_*/
Perlucidibaca_*/
Psychrobacter_*/
### Family Pseudomonadaceae
Azomonas_*/
Azomonotrichon_*/
Azorhizophilus_*/
Azotobacter_*/
Cellvibrio_*/
Chryseomonas_*/
Flavimonas_*/
Mesophilobacter_*/
Pseudomonas_*/
Rhizobacter_*/
Rugamonas_*/
Serpens_*/
#### unclassified Pseudomonadales
Dasania_*/
Oceanicoccus_*/

Eighty-eight (88) closed and 940 draft genomes were downloaded. The complete list of downloaded genomes can be found at the end of this document.

## Pseudo-Pseudomonadales

When examined, it was found that the taxonomic records for three of the downloaded genomes suggested that they were not actually Pseudomonadales. They were,

- Cellvibrio_gilvus_ATCC_13127_uid33853, which is classified as a *Cellulomonas*,
- Pseudomonas_flectens_ATCC_12775_uid204094 which is classified as a *Phaseolibacter*, and
- Pseudomonas_geniculata_N1_uid156719, which is classified as a *Xanthomonadaceae*.

Since the final results were organized around the taxonomic tree of the Pseudomonadales, these three genomes were excluded from further analysis.

## Prodigal Gene Calls

One difficulty with computing orthologs for closed and draft genomes is the uneven quality of the gene annotations. In order to ameliorate this problem, we used Prodigal [1] version 2.60 to compute a *de novo* gene annotation for each genome. In order to assess how much difference this might make to the final computation, we compared the curated annotation for DC3000 against the gene calls produced by Prodigal and Easygene [2]. We found that,

- 3761 times, annotations from the three sets overlap.
- 592 times, the curated and Prodigal annotations overlap.
- 260 times, the Prodigal annotations did not overlap with the others.
- 172 times, the curated annotation did not overlap with the others.
- 28 times, the EasyGene annotation did not overlap with the others.
- 83 times, the Easy Gene and Prodigal annotations overlap.
- 1 time, the curated and EasyGene annotations overlap.

Although there were a number of difference, we found that the Prodigal gene calls to be substantially similar to the curated annotation. If anything, it would appear that Prodigal calls genes more permissively than the DC3000 curator. For our purposes, this is actually an advantage, as it likely to result in more orthologs being found than if the curated annotation were used. And, of course, many of the draft genomes lack a curated annotation, or any annotation at all.

As observed in the full paper, PSPTO_1900 has a ChIP-Seq peak and MEME motif. However, Prodigal did not predict a gene call in the PSPTO_1900 locus. Thus, that gene was not included in the subsequent analysis.

## Reciprical BLAST

The coding sequences from the Prodical gene calls from every genome was BLAST’ed against DC3000’s predicted genes, and vice versa. Version 2.2.29 of NCBI BLAST+ and the following command were used,

blastp -outfmt "6 qseqid sseqid pident length mismatch gapopen
 qstart qend sstart send evalue bitscore score"
 -evalue 1e-6 -searchsp 1750000000000
 -soft_masking true -use_sw_tback
 -query tmp/inputs/GENOME1.faa -db tmp/dbs/GENOME2

Explanation of BLAST+ parameters

- -evalue 1e-6 (Select a cutoff)
- -searchsp 1750000000000 (Normalize the e-values by fixing the search space size)
- -soft_masking true -use_sw_tback (Use soft masking and Smith-Waterman as described in [3])

The output format is equilvant to the std output format (Classic: -m8), except an extra column with the raw score appears rightmost.

## Finding best reciprical hits

A custom script^[[1]](#footnote-28)^ was used to parse the output of each pair of BLAST runs and determine the list of best reciprocal hits.

A pair of gene A and B are best reciprical hits (⇔), iff

- (A ⇔ B) iff (A ⇒ B) and (B ⇒ A)

B is a best hit of A (A ⇒ B) if,

- A hit to B (A→B) is the in output of
- blastp -querty GENOME_OF_A.fna -db GENOME_OF_B
- and the raw bit score of the hit is the maximum raw bit score for all hits of A in GENOME_OF_B.

## Finding orthologs

Each row in S2 Table corresponds to one of the downloaded GENBANK Pseudomonadales genomes. Each column summarizes the orthology analysis for a single gene in DC3000. The first step in this analysis is to compute whether or not the DC3000 gene has an ortholog in the related GENBANK genomes. This is simply done by custom scripts that search for the appropriate “A ⇔ B” gene pair occurs in the best reciprical hits data set. In addition, the DC3000 gene is recorded as its own ortholog. This is necessary, as the results of the DC3000 gene are used to normalize the results from other genomes.

## Analysis of upstream regions

The next step of the analysis is to see of how strong the DC3000 PSPTO_1043 motif (Figure 1 in the paper) is in the sequences upstream of each orthologous gene.

For each column (which corresponds to a single DC3000 gene in the PSPTO_1043 regulon), the genomic sequences upstream of the orthologous genes are extracted from the corresponding genomes. The precise sequence is from δ-250 to δ-1 upstream of the predicted start of the orthologous gene. The value of δ varies from DC3000 genes and is listed below. The upstream sequences are formatted as a FASTA file.

To be clear, a FASTA file of upstream sequences is generated for each DC3000 gene being tested and for each value of δ for that gene.

The sequences in each FASTA file are searched for the PSPTO_1043 motif (Figure 1 in the full paper) using the fimo version 4.9.1 (patch 1) from the MEME package [4]. The following additional parameters were used with fimo

--text --no-qvalue --norc --thresh 1e-1 --bgfile <GENOME.null>

The background model (GENOME.null) is genome specific and is simply the frequency of bases within each genome.

## Summarizing the results

In S2 Table, each row corresponds to a Pseudomonadales genome that was analyzed. Columns M and N list genus/species and GENBANK directory of each genome. Columns B through L encode the taxonomic relationship between the genomes as found in NCBI’s taxonomic database. Column A records the order of the genomes relative to this taxonomic relationship; the original order of the rows to can be restored by sorting the rows on this column in decreasing order. *Pseudomonas* genomes names are color coded for each easier visualization. The color legend can be found on Sheet2 of the spreadsheet.

Column O records the presence (a value of 1.0) or absence (a blank cell) of a PSPTO_1042 ortholog in each genome.

Columns P through AK records the presence (a numeric value) or absence (a blank cell) of an ortholog in each genome for the following genes,

| Column | DC3000 gene | δ |
| --- | --- | --- |
| P | PSPTO_0744 | 0 |
| Q | PSPTO_1043 | 0 |
| R | PSPTO_1121 (phrB) | 0 |
| S | PSPTO_1372 (hopAA1-1) | 0 |
| T | PSPTO_2591 | 0 |
| U | PSPTO_2593 | +3 |
| W | PSPTO_2615 | 0 |
| X | PSPTO_2853 | -174 |
| Z | PSPTO_3893 | 0 |
| AA | PSPTO_3907 | -36 |
| AC | PSPTO_4231 (tctD) | 0 |
| AD | PSPTO_4335 | 0 |
| AE | PSPTO_4530 (katG) | 0 |
| AF | PSPTO_4675 | +9 |
| AH | PSPTO_4702 | 0 |
| AI | PSPTO_4723 | -12 |
| AK | PSPTO_4843 | -6 |

The δ value is the displacement used when extracting the upstream region for motif scanned and was described above. In the cases where a non-zero δ was used, an hidden column shows the results of the analysis for δ=0.

The numerical value reported in each cell is the score of strongest hit to the PSPTO_1043 motif found in that ortholog’s upstream region. The background color of the cell denotes the strength of the hit relative to the strongest hit, which is from DC3000. Dark green denotes the strongest hits, and dark yellow the weakest.

## Downloaded Closed GENBANK Genomes

Acinetobacter_DR1_uid46105/
Acinetobacter_baumannii_1656_2_uid42153/
Acinetobacter_baumannii_AB0057_uid21111/
Acinetobacter_baumannii_AB307_0294_uid30993/
Acinetobacter_baumannii_ACICU_uid17827/
Acinetobacter_baumannii_ATCC_17978_uid17477/
Acinetobacter_baumannii_AYE_uid28921/
Acinetobacter_baumannii_BJAB07104_uid74421/
Acinetobacter_baumannii_BJAB0715_uid74423/
Acinetobacter_baumannii_BJAB0868_uid74425/
Acinetobacter_baumannii_D1279779_uid61919/
Acinetobacter_baumannii_MDR_TJ_uid52959/
Acinetobacter_baumannii_MDR_ZJ06_uid28333/
Acinetobacter_baumannii_TCDC_AB0715_uid62279/
Acinetobacter_baumannii_TYTH_1_uid74551/
Acinetobacter_baumannii_ZW85_1_uid219230/
Acinetobacter_baumannii_uid13001/
Acinetobacter_calcoaceticus_PHEA_2_uid51267/
Acinetobacter_sp_ADP1_uid12352/
Azotobacter_vinelandii_CA6_uid186699/
Azotobacter_vinelandii_CA_uid186698/
Azotobacter_vinelandii_DJ_uid16/
Cellvibrio_gilvus_ATCC_13127_uid33853/
Cellvibrio_japonicus_Ueda107_uid28329/
Moraxella_catarrhalis_RH4_uid46869/
Pseudomonas_FGI182_uid173784/
Pseudomonas_ND6_uid162897/
Pseudomonas_TKP_uid225946/
Pseudomonas_VLB120_uid178195/
Pseudomonas_aeruginosa_B136_33_uid185969/
Pseudomonas_aeruginosa_DK2_uid73815/
Pseudomonas_aeruginosa_LES431_uid222518/
Pseudomonas_aeruginosa_LESB58_uid31101/
Pseudomonas_aeruginosa_M18_uid61423/
Pseudomonas_aeruginosa_MTB_uid225944/
Pseudomonas_aeruginosa_NCGM2_S1_uid73453/
Pseudomonas_aeruginosa_PA1R_uid185336/
Pseudomonas_aeruginosa_PA1_uid185335/
Pseudomonas_aeruginosa_PA7_uid16720/
Pseudomonas_aeruginosa_PAO1_VE13_uid212862/
Pseudomonas_aeruginosa_PAO1_VE2_uid212861/
Pseudomonas_aeruginosa_PAO581_uid209856/
Pseudomonas_aeruginosa_RP73_uid206088/
Pseudomonas_aeruginosa_SCV20265_uid229445/
Pseudomonas_aeruginosa_UCBPP-PA14_uid386/
Pseudomonas_aeruginosa_c7447m_uid209859/
Pseudomonas_aeruginosa_uid331/
Pseudomonas_brassicacearum_NFM421_uid63495/
Pseudomonas_cichorii_JBC1_uid232591/
Pseudomonas_denitrificans_ATCC_13867_uid189431/
Pseudomonas_entomophila_L48_uid16800/
Pseudomonas_fluorescens_A506_uid67529/
Pseudomonas_fluorescens_CHA0_uid78307/
Pseudomonas_fluorescens_F113_uid76715/
Pseudomonas_fluorescens_Pf-5_uid327/
Pseudomonas_fluorescens_Pf0_1_uid12/
Pseudomonas_fluorescens_SBW25_uid31229/
Pseudomonas_fulva_12_X_uid49675/
Pseudomonas_mendocina_NK_01_uid64797/
Pseudomonas_mendocina_ymp_uid17457/
Pseudomonas_monteilii_SB3078_uid231107/
Pseudomonas_monteilii_SB3101_uid231108/
Pseudomonas_poae_RE_1_1_14_uid186492/
Pseudomonas_putida_BIRD_1_uid54033/
Pseudomonas_putida_DOT_T1E_uid167844/
Pseudomonas_putida_F1_uid13909/
Pseudomonas_putida_GB_1_uid17629/
Pseudomonas_putida_H8234_uid202087/
Pseudomonas_putida_HB3267_uid171349/
Pseudomonas_putida_KT2440_uid267/
Pseudomonas_putida_NBRC_14164_uid171050/
Pseudomonas_putida_S16_uid67881/
Pseudomonas_putida_UW4_uid170097/
Pseudomonas_putida_W619_uid17053/
Pseudomonas_resinovorans_NBRC_106553_uid178156/
Pseudomonas_stutzeri_A1501_uid16817/
Pseudomonas_stutzeri_ATCC_17588___LMG_11199_uid68131/
Pseudomonas_stutzeri_CCUG_29243_uid167996/
Pseudomonas_stutzeri_DSM_10701_uid78211/
Pseudomonas_stutzeri_DSM_4166_uid63543/
Pseudomonas_stutzeri_RCH2_uid60029/
Pseudomonas_syringae_phaseolicola_1448A_uid12416/
Pseudomonas_syringae_pv_B728a_uid323/
Pseudomonas_syringae_tomato_DC3000_uid359/
Psychrobacter_G_uid209108/
Psychrobacter_PRwf-1_uid15759/
Psychrobacter_arcticum_273-4_uid9633/
Psychrobacter_cryohalolentis_K5_uid13920/

## Downloaded Draft GENBANK Genomes

Acinetobacter_528_uid172719/
Acinetobacter_6013113_uid33017/
Acinetobacter_6013150_uid33071/
Acinetobacter_6014059_uid33073/
Acinetobacter_ANC_3789_uid183313/
Acinetobacter_ANC_3811_uid183273/
Acinetobacter_ANC_3862_uid183238/
Acinetobacter_ANC_3880_uid183223/
Acinetobacter_ANC_3929_uid183247/
Acinetobacter_ANC_3994_uid183332/
Acinetobacter_ANC_4050_uid183269/
Acinetobacter_ANC_4052_uid183267/
Acinetobacter_ANC_4105_uid183242/
Acinetobacter_ATCC_27244_uid30949/
Acinetobacter_CAG_196_uid206614/
Acinetobacter_CIP_101934_uid183237/
Acinetobacter_CIP_101966_uid183229/
Acinetobacter_CIP_102082_uid183308/
Acinetobacter_CIP_102129_uid183311/
Acinetobacter_CIP_102136_uid183231/
Acinetobacter_CIP_102143_uid183222/
Acinetobacter_CIP_102159_uid183312/
Acinetobacter_CIP_102529_uid183310/
Acinetobacter_CIP_102637_uid183305/
Acinetobacter_CIP_110321_uid183234/
Acinetobacter_CIP_51_11_uid183232/
Acinetobacter_CIP_53_82_uid183243/
Acinetobacter_CIP_56_2_uid183304/
Acinetobacter_CIP_64_2_uid183233/
Acinetobacter_CIP_64_7_uid183228/
Acinetobacter_CIP_70_18_uid183240/
Acinetobacter_CIP_A162_uid183333/
Acinetobacter_CIP_A165_uid183329/
Acinetobacter_GG2_uid171723/
Acinetobacter_HA_uid158435/
Acinetobacter_MDS7A_uid186610/
Acinetobacter_NBRC_100985_uid71037/
Acinetobacter_NCTC_10304_uid62435/
Acinetobacter_NCTC_7422_uid62433/
Acinetobacter_NIPH_1847_uid183236/
Acinetobacter_NIPH_1859_uid183227/
Acinetobacter_NIPH_1867_uid183239/
Acinetobacter_NIPH_2036_uid183245/
Acinetobacter_NIPH_2100_uid183225/
Acinetobacter_NIPH_2168_uid183230/
Acinetobacter_NIPH_2171_uid183235/
Acinetobacter_NIPH_236_uid183330/
Acinetobacter_NIPH_284_uid183246/
Acinetobacter_NIPH_298_uid183241/
Acinetobacter_NIPH_3623_uid183226/
Acinetobacter_NIPH_542_uid183224/
Acinetobacter_NIPH_713_uid183244/
Acinetobacter_NIPH_758_uid183309/
Acinetobacter_NIPH_809_uid183331/
Acinetobacter_NIPH_817_uid183306/
Acinetobacter_NIPH_899_uid183307/
Acinetobacter_NIPH_973_uid183323/
Acinetobacter_P8_3_8_uid66629/
Acinetobacter_RUH2624_uid38511/
Acinetobacter_SH024_uid41947/
Acinetobacter_TG19627_uid172720/
Acinetobacter_TG2027_uid172721/
Acinetobacter_TG27347_uid172701/
Acinetobacter_WC_743_uid53455/
Acinetobacter_baumannii_107m_uid222617/
Acinetobacter_baumannii_1605_uid209530/
Acinetobacter_baumannii_3909_uid52397/
Acinetobacter_baumannii_3990_uid52395/
Acinetobacter_baumannii_4190_uid52399/
Acinetobacter_baumannii_48055_uid189305/
Acinetobacter_baumannii_4857_uid77019/
Acinetobacter_baumannii_5075_uid77021/
Acinetobacter_baumannii_5256_uid77023/
Acinetobacter_baumannii_53264_uid171521/
Acinetobacter_baumannii_5711_uid77025/
Acinetobacter_baumannii_A118_uid60111/
Acinetobacter_baumannii_AA_014_uid53475/
Acinetobacter_baumannii_AB056_uid43427/
Acinetobacter_baumannii_AB058_uid43429/
Acinetobacter_baumannii_AB059_uid43431/
Acinetobacter_baumannii_AB10I9_uid208611/
Acinetobacter_baumannii_AB18G7_uid208613/
Acinetobacter_baumannii_AB1H8_uid172835/
Acinetobacter_baumannii_AB210_uid52391/
Acinetobacter_baumannii_AB405E4_uid172837/
Acinetobacter_baumannii_AB4A3_uid172836/
Acinetobacter_baumannii_AB900_uid30995/
Acinetobacter_baumannii_ABIsac_ColiR_uid184938/
Acinetobacter_baumannii_ABIsac_ColiS_uid185027/
Acinetobacter_baumannii_ABNIH10_uid73913/
Acinetobacter_baumannii_ABNIH11_uid73915/
Acinetobacter_baumannii_ABNIH13_uid73919/
Acinetobacter_baumannii_ABNIH14_uid73921/
Acinetobacter_baumannii_ABNIH15_uid73923/
Acinetobacter_baumannii_ABNIH16_uid73925/
Acinetobacter_baumannii_ABNIH17_uid73927/
Acinetobacter_baumannii_ABNIH18_uid73929/
Acinetobacter_baumannii_ABNIH19_uid73931/
Acinetobacter_baumannii_ABNIH1_uid63335/
Acinetobacter_baumannii_ABNIH20_uid73933/
Acinetobacter_baumannii_ABNIH22_uid73937/
Acinetobacter_baumannii_ABNIH23_uid73939/
Acinetobacter_baumannii_ABNIH24_uid73941/
Acinetobacter_baumannii_ABNIH25_uid172976/
Acinetobacter_baumannii_ABNIH26_uid172977/
Acinetobacter_baumannii_ABNIH2_uid63337/
Acinetobacter_baumannii_ABNIH3_uid63339/
Acinetobacter_baumannii_ABNIH4_uid63341/
Acinetobacter_baumannii_ABNIH5_uid73903/
Acinetobacter_baumannii_ABNIH6_uid73905/
Acinetobacter_baumannii_ABNIH7_uid73907/
Acinetobacter_baumannii_AB_1536_8_uid172655/
Acinetobacter_baumannii_AB_1582_8_uid172656/
Acinetobacter_baumannii_AB_1583_8_uid172657/
Acinetobacter_baumannii_AB_1594_8_uid172658/
Acinetobacter_baumannii_AB_1595_8_uid172659/
Acinetobacter_baumannii_AB_1649_8_uid172660/
Acinetobacter_baumannii_AB_1650_8_uid172661/
Acinetobacter_baumannii_AB_1766_8_uid172662/
Acinetobacter_baumannii_AB_2007_09_110_01_7_uid172663/
Acinetobacter_baumannii_AB_2007_16_25_01_7_uid172664/
Acinetobacter_baumannii_AB_2007_16_27_01_uid172665/
Acinetobacter_baumannii_AB_2008_15_34_7_uid172666/
Acinetobacter_baumannii_AB_2008_15_45_uid172667/
Acinetobacter_baumannii_AB_2008_15_52_uid172668/
Acinetobacter_baumannii_AB_2008_15_69_uid172669/
Acinetobacter_baumannii_AB_2008_15_70_uid172670/
Acinetobacter_baumannii_AB_2008_15_71_uid172671/
Acinetobacter_baumannii_AB_2008_23_01_01_7_uid172672/
Acinetobacter_baumannii_AB_2008_23_07_01_7_uid172673/
Acinetobacter_baumannii_AB_2009_04_01_7_uid172674/
Acinetobacter_baumannii_AB_2009_04_02_7_uid172675/
Acinetobacter_baumannii_AB_515_8_uid172676/
Acinetobacter_baumannii_AB_908_12_uid172677/
Acinetobacter_baumannii_AB_908_13_uid172678/
Acinetobacter_baumannii_AB_908_14_7_uid172679/
Acinetobacter_baumannii_AB_909_01_7_uid172680/
Acinetobacter_baumannii_AB_909_02_7_uid172681/
Acinetobacter_baumannii_AB_909_05_uid172682/
Acinetobacter_baumannii_AB_909_14_7_uid172683/
Acinetobacter_baumannii_AB_TG19617_uid172685/
Acinetobacter_baumannii_AB_TG2018_uid172686/
Acinetobacter_baumannii_AB_TG2022_uid172687/
Acinetobacter_baumannii_AB_TG2023_uid172688/
Acinetobacter_baumannii_AB_TG2026_uid172689/
Acinetobacter_baumannii_AB_TG2028_uid172690/
Acinetobacter_baumannii_AB_TG2030_uid172691/
Acinetobacter_baumannii_AB_TG2031_uid172692/
Acinetobacter_baumannii_AB_TG2032_uid172693/
Acinetobacter_baumannii_AB_TG2631_uid172694/
Acinetobacter_baumannii_AB_TG27323_uid172695/
Acinetobacter_baumannii_AB_TG27327_uid172696/
Acinetobacter_baumannii_AB_TG27331_uid172697/
Acinetobacter_baumannii_AB_TG27335_uid172698/
Acinetobacter_baumannii_AB_TG27339_uid172699/
Acinetobacter_baumannii_AB_TG27343_uid172700/
Acinetobacter_baumannii_AB_TG5064_uid172702/
Acinetobacter_baumannii_AC12_uid168112/
Acinetobacter_baumannii_AC30_uid173033/
Acinetobacter_baumannii_ANC_4097_uid183250/
Acinetobacter_baumannii_ATCC_19606___CIP_70_34_uid183249/
Acinetobacter_baumannii_ATCC_19606_uid38509/
Acinetobacter_baumannii_Ab11111_uid157163/
Acinetobacter_baumannii_Ab33333_uid157167/
Acinetobacter_baumannii_Ab44444_uid157169/
Acinetobacter_baumannii_BZICU_2_uid170599/
Acinetobacter_baumannii_CI77_uid206987/
Acinetobacter_baumannii_CI78_uid206973/
Acinetobacter_baumannii_CI79_uid206979/
Acinetobacter_baumannii_CI86_uid206988/
Acinetobacter_baumannii_Canada_BC1_uid53447/
Acinetobacter_baumannii_Canada_BC_5_uid53389/
Acinetobacter_baumannii_DU202_uid213256/
Acinetobacter_baumannii_EGD_HP18_uid211933/
Acinetobacter_baumannii_IS_116_uid53423/
Acinetobacter_baumannii_IS_123_uid53397/
Acinetobacter_baumannii_IS_143_uid53421/
Acinetobacter_baumannii_IS_235_uid53409/
Acinetobacter_baumannii_IS_251_uid53411/
Acinetobacter_baumannii_IS_58_uid53427/
Acinetobacter_baumannii_M2_uid206997/
Acinetobacter_baumannii_MDR_MMC4_uid231087/
Acinetobacter_baumannii_MRY09_0642_uid231360/
Acinetobacter_baumannii_MRY10_0558_uid231361/
Acinetobacter_baumannii_MRY12_0277_uid231362/
Acinetobacter_baumannii_MSP4_16_uid185251/
Acinetobacter_baumannii_NIPH_1362_uid183320/
Acinetobacter_baumannii_NIPH_146_uid183317/
Acinetobacter_baumannii_NIPH_1669_uid183321/
Acinetobacter_baumannii_NIPH_1734_uid183314/
Acinetobacter_baumannii_NIPH_190_uid183300/
Acinetobacter_baumannii_NIPH_201_uid183260/
Acinetobacter_baumannii_NIPH_2061_uid183315/
Acinetobacter_baumannii_NIPH_24_uid183334/
Acinetobacter_baumannii_NIPH_290_uid183252/
Acinetobacter_baumannii_NIPH_329_uid183257/
Acinetobacter_baumannii_NIPH_335_uid183258/
Acinetobacter_baumannii_NIPH_410_uid183248/
Acinetobacter_baumannii_NIPH_527_uid183259/
Acinetobacter_baumannii_NIPH_528_uid183254/
Acinetobacter_baumannii_NIPH_601_uid183256/
Acinetobacter_baumannii_NIPH_60_uid183299/
Acinetobacter_baumannii_NIPH_615_uid183316/
Acinetobacter_baumannii_NIPH_67_uid183255/
Acinetobacter_baumannii_NIPH_70_uid183253/
Acinetobacter_baumannii_NIPH_80_uid183251/
Acinetobacter_baumannii_Naval_113_uid53439/
Acinetobacter_baumannii_Naval_13_uid53407/
Acinetobacter_baumannii_Naval_17_uid53391/
Acinetobacter_baumannii_Naval_18_uid53393/
Acinetobacter_baumannii_Naval_21_uid53445/
Acinetobacter_baumannii_Naval_2_uid53443/
Acinetobacter_baumannii_Naval_57_uid53453/
Acinetobacter_baumannii_Naval_72_uid53415/
Acinetobacter_baumannii_Naval_78_uid53473/
Acinetobacter_baumannii_Naval_81_uid53395/
Acinetobacter_baumannii_Naval_82_uid53441/
Acinetobacter_baumannii_Naval_83_uid53417/
Acinetobacter_baumannii_OIFC0162_uid53413/
Acinetobacter_baumannii_OIFC021_uid53457/
Acinetobacter_baumannii_OIFC032_uid53379/
Acinetobacter_baumannii_OIFC035_uid53451/
Acinetobacter_baumannii_OIFC047_uid53467/
Acinetobacter_baumannii_OIFC065_uid53465/
Acinetobacter_baumannii_OIFC074_uid53401/
Acinetobacter_baumannii_OIFC087_uid53459/
Acinetobacter_baumannii_OIFC098_uid53403/
Acinetobacter_baumannii_OIFC099_uid53461/
Acinetobacter_baumannii_OIFC109_uid53383/
Acinetobacter_baumannii_OIFC110_uid53419/
Acinetobacter_baumannii_OIFC111_uid53471/
Acinetobacter_baumannii_OIFC137_uid53377/
Acinetobacter_baumannii_OIFC143_uid53385/
Acinetobacter_baumannii_OIFC180_uid53405/
Acinetobacter_baumannii_OIFC189_uid53387/
Acinetobacter_baumannii_OIFC338_uid53469/
Acinetobacter_baumannii_PR07_uid185400/
Acinetobacter_baumannii_Perm_uid209318/
Acinetobacter_baumannii_TG00314_uid196253/
Acinetobacter_baumannii_TG02011_uid196254/
Acinetobacter_baumannii_TG02017_uid196255/
Acinetobacter_baumannii_TG07725_uid196256/
Acinetobacter_baumannii_TG15233_uid196257/
Acinetobacter_baumannii_TG15234_uid196258/
Acinetobacter_baumannii_TG15236_uid196259/
Acinetobacter_baumannii_TG15237_uid196260/
Acinetobacter_baumannii_TG15238_uid196261/
Acinetobacter_baumannii_TG15239_uid196262/
Acinetobacter_baumannii_TG15240_uid196263/
Acinetobacter_baumannii_TG15241_uid196264/
Acinetobacter_baumannii_TG15242_uid196265/
Acinetobacter_baumannii_TG19582_uid172703/
Acinetobacter_baumannii_TG2012_uid196266/
Acinetobacter_baumannii_TG2013_uid196267/
Acinetobacter_baumannii_TG2014_uid196268/
Acinetobacter_baumannii_TG20277_uid196269/
Acinetobacter_baumannii_TG20546_uid196270/
Acinetobacter_baumannii_TG22110_uid196273/
Acinetobacter_baumannii_TG22112_uid196274/
Acinetobacter_baumannii_TG22142_uid196275/
Acinetobacter_baumannii_TG22146_uid196276/
Acinetobacter_baumannii_TG22148_uid196277/
Acinetobacter_baumannii_TG22150_uid196278/
Acinetobacter_baumannii_TG22190_uid196279/
Acinetobacter_baumannii_TG22192_uid196280/
Acinetobacter_baumannii_TG22194_uid196281/
Acinetobacter_baumannii_TG22196_uid196282/
Acinetobacter_baumannii_TG22198_uid196283/
Acinetobacter_baumannii_TG22202_uid196284/
Acinetobacter_baumannii_TG22204_uid196285/
Acinetobacter_baumannii_TG22212_uid196286/
Acinetobacter_baumannii_TG22214_uid196287/
Acinetobacter_baumannii_TG22332_uid196288/
Acinetobacter_baumannii_TG22336_uid196289/
Acinetobacter_baumannii_TG27295_uid196290/
Acinetobacter_baumannii_TG27299_uid196291/
Acinetobacter_baumannii_TG27307_uid196292/
Acinetobacter_baumannii_TG27311_uid196293/
Acinetobacter_baumannii_TG27315_uid196294/
Acinetobacter_baumannii_TG27319_uid196295/
Acinetobacter_baumannii_TG27371_uid196296/
Acinetobacter_baumannii_TG27379_uid196297/
Acinetobacter_baumannii_TG27383_uid196298/
Acinetobacter_baumannii_TG27387_uid196299/
Acinetobacter_baumannii_TG27391_uid196300/
Acinetobacter_baumannii_TG27395_uid196301/
Acinetobacter_baumannii_TG27399_uid196302/
Acinetobacter_baumannii_TG27407_uid196303/
Acinetobacter_baumannii_TG27411_uid196304/
Acinetobacter_baumannii_UH0207_uid218744/
Acinetobacter_baumannii_UH0707_uid218745/
Acinetobacter_baumannii_UH0807_uid218746/
Acinetobacter_baumannii_UH10007_uid218748/
Acinetobacter_baumannii_UH1007_uid218747/
Acinetobacter_baumannii_UH10107_uid218749/
Acinetobacter_baumannii_UH10707_uid218750/
Acinetobacter_baumannii_UH10707_uid227030/
Acinetobacter_baumannii_UH11608_uid218751/
Acinetobacter_baumannii_UH12208_uid218752/
Acinetobacter_baumannii_UH12308_uid218753/
Acinetobacter_baumannii_UH12408_uid218754/
Acinetobacter_baumannii_UH12808_uid218755/
Acinetobacter_baumannii_UH13908_uid218756/
Acinetobacter_baumannii_UH14508_uid218757/
Acinetobacter_baumannii_UH15208_uid218758/
Acinetobacter_baumannii_UH16008_uid218759/
Acinetobacter_baumannii_UH16108_uid218760/
Acinetobacter_baumannii_UH16208_uid218761/
Acinetobacter_baumannii_UH18608_uid218762/
Acinetobacter_baumannii_UH19608_uid218763/
Acinetobacter_baumannii_UH19908_uid218764/
Acinetobacter_baumannii_UH20108_uid218765/
Acinetobacter_baumannii_UH2107_uid218766/
Acinetobacter_baumannii_UH22908_uid218767/
Acinetobacter_baumannii_UH2307_uid218768/
Acinetobacter_baumannii_UH2707_uid218769/
Acinetobacter_baumannii_UH2907_uid218770/
Acinetobacter_baumannii_UH3807_uid218771/
Acinetobacter_baumannii_UH5107_uid218772/
Acinetobacter_baumannii_UH5207_uid218773/
Acinetobacter_baumannii_UH5307_uid218774/
Acinetobacter_baumannii_UH5707_uid218775/
Acinetobacter_baumannii_UH6107_uid218776/
Acinetobacter_baumannii_UH6207_uid218777/
Acinetobacter_baumannii_UH6507_uid218778/
Acinetobacter_baumannii_UH6907_uid218779/
Acinetobacter_baumannii_UH7007_uid218780/
Acinetobacter_baumannii_UH7607_uid218781/
Acinetobacter_baumannii_UH7707_uid218782/
Acinetobacter_baumannii_UH7807_uid218783/
Acinetobacter_baumannii_UH7907_uid218784/
Acinetobacter_baumannii_UH8107_uid218785/
Acinetobacter_baumannii_UH8407_uid218786/
Acinetobacter_baumannii_UH8707_uid218787/
Acinetobacter_baumannii_UH8807_uid218788/
Acinetobacter_baumannii_UH8907_uid218789/
Acinetobacter_baumannii_UH9007_uid218790/
Acinetobacter_baumannii_UH9707_uid218791/
Acinetobacter_baumannii_UH9907_uid218792/
Acinetobacter_baumannii_UH9907_uid227028/
Acinetobacter_baumannii_UMB001_uid60843/
Acinetobacter_baumannii_UMB002_uid60845/
Acinetobacter_baumannii_UMB003_uid60847/
Acinetobacter_baumannii_W6976_uid62419/
Acinetobacter_baumannii_W7282_uid62421/
Acinetobacter_baumannii_WC_136_uid53431/
Acinetobacter_baumannii_WC_141_uid53429/
Acinetobacter_baumannii_WC_323_uid53435/
Acinetobacter_baumannii_WC_348_uid53437/
Acinetobacter_baumannii_WC_487_uid53433/
Acinetobacter_baumannii_WC_692_uid53425/
Acinetobacter_baumannii_WC_A_694_uid53449/
Acinetobacter_baumannii_WC_A_92_uid53463/
Acinetobacter_baumannii_WM99c_uid61917/
Acinetobacter_baumannii_ZWS1122_uid172873/
Acinetobacter_baumannii_ZWS1219_uid172947/
Acinetobacter_baylyi_DSM_14961___CIP_107474_uid183290/
Acinetobacter_baylyi_TG19579_uid172684/
Acinetobacter_beijerinckii_ANC_3835_uid183272/
Acinetobacter_beijerinckii_CIP_110307_uid183271/
Acinetobacter_bereziniae_CIP_70_12_uid183276/
Acinetobacter_bereziniae_LMG_1003_uid62411/
Acinetobacter_bereziniae_NIPH_3_uid183301/
Acinetobacter_bouvetii_DSM_14964___CIP_107468_uid169762/
Acinetobacter_bouvetii_DSM_14964___CIP_107468_uid183279/
Acinetobacter_brisouii_ANC_4119_uid183292/
Acinetobacter_brisouii_CIP_110357_uid217359/
Acinetobacter_calcoaceticus_ANC_3680_uid183275/
Acinetobacter_calcoaceticus_DSM_30006___CIP_81_8_uid183274/
Acinetobacter_calcoaceticus_DSM_30006_uid62423/
Acinetobacter_calcoaceticus_NIPH_13_uid183335/
Acinetobacter_calcoaceticus_RUH2202_uid38337/
Acinetobacter_calcoaceticus_TG19585_uid172704/
Acinetobacter_calcoaceticus_TG19588_uid172705/
Acinetobacter_calcoaceticus_TG19593_uid172706/
Acinetobacter_calcoaceticus_anitratus_XM1570_uid173032/
Acinetobacter_genomosp__13TU_NCTC_8102_uid62413/
Acinetobacter_genomosp__3_DSM_21653_uid62415/
Acinetobacter_genomosp__3_DSM_9306_uid62425/
Acinetobacter_gerneri_DSM_14967___CIP_107464_uid183298/
Acinetobacter_gerneri_MTCC9824_uid201032/
Acinetobacter_guillouiae_CIP_63_46_uid183319/
Acinetobacter_guillouiae_MSP4_18_uid201034/
Acinetobacter_guillouiae_NIPH_991_uid183302/
Acinetobacter_gyllenbergii_CIP_110306_uid183295/
Acinetobacter_gyllenbergii_MTCC11365_uid201036/
Acinetobacter_gyllenbergii_NIPH_230_uid183325/
Acinetobacter_haemolyticus_ATCC_19194_uid43187/
Acinetobacter_haemolyticus_CIP_64_3_uid183265/
Acinetobacter_haemolyticus_MTCC_9819_uid201356/
Acinetobacter_haemolyticus_NIPH_261_uid183264/
Acinetobacter_haemolyticus_TG19599_uid172708/
Acinetobacter_haemolyticus_TG19602_uid172709/
Acinetobacter_haemolyticus_TG21157_uid172710/
Acinetobacter_indicus_ANC_4215_uid183294/
Acinetobacter_indicus_CIP_110367_uid217357/
Acinetobacter_johnsonii_ANC_3681_uid183284/
Acinetobacter_johnsonii_CIP_64_6_uid183324/
Acinetobacter_johnsonii_SH046_uid38339/
Acinetobacter_johnsonii_TG19605_uid172711/
Acinetobacter_johnsonii_TG19625_uid172712/
Acinetobacter_junii_CIP_107470_uid183291/
Acinetobacter_junii_CIP_64_5_uid183286/
Acinetobacter_junii_MTCC11364_uid201035/
Acinetobacter_junii_NIPH_182_uid183287/
Acinetobacter_junii_SH205_uid38341/
Acinetobacter_junii_TG19608_uid172713/
Acinetobacter_lwoffii_CIP_70_31_uid183262/
Acinetobacter_lwoffii_NCTC_5866___CIP_64_10_uid183263/
Acinetobacter_lwoffii_NCTC_5866_uid62417/
Acinetobacter_lwoffii_NIPH_478_uid183261/
Acinetobacter_lwoffii_NIPH_512_uid219244/
Acinetobacter_lwoffii_NIPH_715_uid183318/
Acinetobacter_lwoffii_SH145_uid38343/
Acinetobacter_lwoffii_TG19636_uid172714/
Acinetobacter_lwoffii_WJ10621_uid68097/
Acinetobacter_nectaris_CIP_110549_uid217360/
Acinetobacter_nosocomialis_28F_uid222615/
Acinetobacter_nosocomialis_Ab22222_uid164597/
Acinetobacter_nosocomialis_NIPH_2119_uid183322/
Acinetobacter_nosocomialis_NIPH_386_uid183296/
Acinetobacter_nosocomialis_TG19596_uid172707/
Acinetobacter_nosocomialis_TG21145_uid172715/
Acinetobacter_oleivorans_CIP_110421_uid217358/
Acinetobacter_parvus_DSM_16617___CIP_108168_uid183326/
Acinetobacter_parvus_DSM_16617_uid62427/
Acinetobacter_parvus_NIPH_1103_uid183327/
Acinetobacter_pittii_42F_uid222618/
Acinetobacter_pittii_ANC_3678_uid183268/
Acinetobacter_pittii_CIP_70_29_uid183266/
Acinetobacter_pittii_D499_uid73129/
Acinetobacter_pittii_TG6411_uid172716/
Acinetobacter_radioresistens_DSM_6976___NBRC_102413_CIP_103788_uid183277/
Acinetobacter_radioresistens_DSM_6976___NBRC_102413_uid62429/
Acinetobacter_radioresistens_DSM_6976___NBRC_102413_uid84207/
Acinetobacter_radioresistens_NIPH_2130_uid183278/
Acinetobacter_radioresistens_SH164_uid38345/
Acinetobacter_radioresistens_SK82_uid34081/
Acinetobacter_radioresistens_TG02010_uid172717/
Acinetobacter_radioresistens_WC_A_157_uid53399/
Acinetobacter_rudis_CIP_110305_uid183283/
Acinetobacter_schindleri_CIP_107287_uid183293/
Acinetobacter_schindleri_NIPH_900_uid183303/
Acinetobacter_schindleri_TG19614_uid172718/
Acinetobacter_soli_CIP_110264_uid183289/
Acinetobacter_soli_NIPH_2899_uid183288/
Acinetobacter_tandoii_DSM_14970___CIP_107469_uid183270/
Acinetobacter_tjernbergiae_DSM_14971___CIP_107465_uid174971/
Acinetobacter_tjernbergiae_DSM_14971___CIP_107465_uid183328/
Acinetobacter_towneri_DSM_14962___CIP_107472_uid183285/
Acinetobacter_ursingii_ANC_3649_uid183280/
Acinetobacter_ursingii_DSM_16037___CIP_107286_uid183282/
Acinetobacter_ursingii_DSM_16037_uid62431/
Acinetobacter_ursingii_NIPH_706_uid183281/
Acinetobacter_venetianus_RAG_1___CIP_110063_uid167043/
Acinetobacter_venetianus_RAG_1___CIP_110063_uid183297/
Acinetobacter_venetianus_VE_C3_uid168177/
Cellvibrio_BR_uid81229/
Dasania_marina_DSM_21967_uid169739/
Enhydrobacter_aerosaccus_SK60_uid31335/
Moraxella_boevrei_DSM_14165_uid165407/
Moraxella_caprae_DSM_19149_uid198543/
Moraxella_catarrhalis_101P30B1_uid49925/
Moraxella_catarrhalis_103P14B1_uid49911/
Moraxella_catarrhalis_46P47B1_uid49913/
Moraxella_catarrhalis_7169_uid49907/
Moraxella_catarrhalis_BC1_uid49917/
Moraxella_catarrhalis_BC7_uid49919/
Moraxella_catarrhalis_BC8_uid49921/
Moraxella_catarrhalis_CO72_uid49923/
Moraxella_catarrhalis_O35E_uid49927/
Moraxella_catarrhalis_RH4_uid175869/
Moraxella_catarrhalis_uid49915/
Moraxella_macacae_0408225_uid174162/
Perlucidibaca_piscinae_DSM_21586_uid182434/
Pseudomonas_103_uid215329/
Pseudomonas_2_1_26_uid40037/
Pseudomonas_2_92_2010__uid224560/
Pseudomonas_313_uid175607/
Pseudomonas_35MFCvi1_1_uid182410/
Pseudomonas_45MFCol3_1_uid185298/
Pseudomonas_Ag1_uid168001/
Pseudomonas_CBZ_4_uid170785/
Pseudomonas_CF149_uid59591/
Pseudomonas_CF150_uid59593/
Pseudomonas_CF161_uid59595/
Pseudomonas_CFII64_uid59597/
Pseudomonas_CFII68_uid59599/
Pseudomonas_CFT9_uid59601/
Pseudomonas_Chol1_uid174132/
Pseudomonas_G5_2012__uid183186/
Pseudomonas_GM102_uid83065/
Pseudomonas_GM16_uid83067/
Pseudomonas_GM17_uid83069/
Pseudomonas_GM18_uid83071/
Pseudomonas_GM21_uid83073/
Pseudomonas_GM24_uid83075/
Pseudomonas_GM25_uid83077/
Pseudomonas_GM30_uid83079/
Pseudomonas_GM33_uid83081/
Pseudomonas_GM41_2012__uid83083/
Pseudomonas_GM48_uid83085/
Pseudomonas_GM49_uid83087/
Pseudomonas_GM50_uid83089/
Pseudomonas_GM55_uid83091/
Pseudomonas_GM60_uid83093/
Pseudomonas_GM67_uid83095/
Pseudomonas_GM74_uid83097/
Pseudomonas_GM78_uid83099/
Pseudomonas_GM79_uid83101/
Pseudomonas_GM80_uid83103/
Pseudomonas_GM84_uid83105/
Pseudomonas_HPB0071_uid169470/
Pseudomonas_HYS_uid89717/
Pseudomonas_JGI_0001012_A11_uid190848/
Pseudomonas_LAIL14HWK12_I12_uid179472/
Pseudomonas_LAIL14HWK12_I5_uid182468/
Pseudomonas_LAIL14HWK12_I6_uid179477/
Pseudomonas_LAIL14HWK12_I7_uid179456/
Pseudomonas_LAIL14HWK12_I9_uid179473/
Pseudomonas_LAMO17WK12_I2_uid179453/
Pseudomonas_LAMO17WK12_I4_uid179455/
Pseudomonas_M1_uid62721/
Pseudomonas_M47T1_uid162465/
Pseudomonas_MOIL14HWK12_I1_uid79067/
Pseudomonas_MOIL14HWK12_I2_uid79069/
Pseudomonas_P179_uid169471/
Pseudomonas_P818_uid204965/
Pseudomonas_PAMC_25886_uid78809/
Pseudomonas_PAMC_26793_uid176955/
Pseudomonas_R62_uid82847/
Pseudomonas_R81_uid82849/
Pseudomonas_S13_1_2_uid171728/
Pseudomonas_S9_uid66201/
Pseudomonas_TJI_51_uid61865/
Pseudomonas_TX1_uid173805/
Pseudomonas_UK4_uid37861/
Pseudomonas_URIL14HWK12_I4_uid186457/
Pseudomonas_URIL14HWK12_I6_uid186475/
Pseudomonas_URIL14HWK12_I7_uid186464/
Pseudomonas_URMO17WK12_I11_uid186473/
Pseudomonas_URMO17WK12_I12_uid179452/
Pseudomonas_URMO17WK12_I3_uid186470/
Pseudomonas_URMO17WK12_I4_uid186474/
Pseudomonas_aeruginosa_138244_uid62723/
Pseudomonas_aeruginosa_152504_uid62725/
Pseudomonas_aeruginosa_18A_uid189459/
Pseudomonas_aeruginosa_19660_uid219858/
Pseudomonas_aeruginosa_19BR_uid70773/
Pseudomonas_aeruginosa_213BR_uid70775/
Pseudomonas_aeruginosa_2192_uid16171/
Pseudomonas_aeruginosa_39016_uid49667/
Pseudomonas_aeruginosa_6077_uid219859/
Pseudomonas_aeruginosa_62_uid219845/
Pseudomonas_aeruginosa_9BR_uid68315/
Pseudomonas_aeruginosa_AES_1R_uid64619/
Pseudomonas_aeruginosa_AH16_uid170163/
Pseudomonas_aeruginosa_ATCC_14886_uid89671/
Pseudomonas_aeruginosa_ATCC_15442_uid228962/
Pseudomonas_aeruginosa_ATCC_25324_uid89673/
Pseudomonas_aeruginosa_ATCC_700888_uid89675/
Pseudomonas_aeruginosa_B3_1811_uid214882/
Pseudomonas_aeruginosa_B3_208_uid214878/
Pseudomonas_aeruginosa_B3_20M_uid214880/
Pseudomonas_aeruginosa_B3_CFI_uid214879/
Pseudomonas_aeruginosa_BK1_uid236406/
Pseudomonas_aeruginosa_BL01_uid219903/
Pseudomonas_aeruginosa_BL02_uid219905/
Pseudomonas_aeruginosa_BL03_uid219906/
Pseudomonas_aeruginosa_BL04_uid219907/
Pseudomonas_aeruginosa_BL05_uid219908/
Pseudomonas_aeruginosa_BL06_uid219909/
Pseudomonas_aeruginosa_BL07_uid219910/
Pseudomonas_aeruginosa_BL08_uid219911/
Pseudomonas_aeruginosa_BL09_uid219912/
Pseudomonas_aeruginosa_BL10_uid219913/
Pseudomonas_aeruginosa_BL11_uid219914/
Pseudomonas_aeruginosa_BL12_uid219915/
Pseudomonas_aeruginosa_BL13_uid219916/
Pseudomonas_aeruginosa_BL14_uid219917/
Pseudomonas_aeruginosa_BL15_uid219918/
Pseudomonas_aeruginosa_BL16_uid219919/
Pseudomonas_aeruginosa_BL17_uid219920/
Pseudomonas_aeruginosa_BL18_uid219921/
Pseudomonas_aeruginosa_BL19_uid219922/
Pseudomonas_aeruginosa_BL20_uid219923/
Pseudomonas_aeruginosa_BL21_uid219924/
Pseudomonas_aeruginosa_BL22_uid219925/
Pseudomonas_aeruginosa_BL23_uid219926/
Pseudomonas_aeruginosa_BL24_uid219927/
Pseudomonas_aeruginosa_BL25_uid219928/
Pseudomonas_aeruginosa_BWHPSA001_uid219862/
Pseudomonas_aeruginosa_BWHPSA002_uid219863/
Pseudomonas_aeruginosa_BWHPSA003_uid219864/
Pseudomonas_aeruginosa_BWHPSA004_uid219865/
Pseudomonas_aeruginosa_BWHPSA005_uid219866/
Pseudomonas_aeruginosa_BWHPSA006_uid219867/
Pseudomonas_aeruginosa_BWHPSA007_uid219868/
Pseudomonas_aeruginosa_BWHPSA008_uid219869/
Pseudomonas_aeruginosa_BWHPSA009_uid219870/
Pseudomonas_aeruginosa_BWHPSA010_uid219871/
Pseudomonas_aeruginosa_BWHPSA011_uid219872/
Pseudomonas_aeruginosa_BWHPSA012_uid219873/
Pseudomonas_aeruginosa_BWHPSA013_uid219874/
Pseudomonas_aeruginosa_BWHPSA014_uid219875/
Pseudomonas_aeruginosa_BWHPSA015_uid219876/
Pseudomonas_aeruginosa_BWHPSA016_uid219877/
Pseudomonas_aeruginosa_BWHPSA017_uid219878/
Pseudomonas_aeruginosa_BWHPSA018_uid219879/
Pseudomonas_aeruginosa_BWHPSA019_uid219880/
Pseudomonas_aeruginosa_BWHPSA020_uid219881/
Pseudomonas_aeruginosa_BWHPSA021_uid219882/
Pseudomonas_aeruginosa_BWHPSA022_uid219883/
Pseudomonas_aeruginosa_BWHPSA023_uid219884/
Pseudomonas_aeruginosa_BWHPSA024_uid219885/
Pseudomonas_aeruginosa_BWHPSA025_uid219886/
Pseudomonas_aeruginosa_BWHPSA026_uid219887/
Pseudomonas_aeruginosa_BWHPSA027_uid219888/
Pseudomonas_aeruginosa_BWHPSA028_uid219889/
Pseudomonas_aeruginosa_BWHPSA037_uid219890/
Pseudomonas_aeruginosa_BWHPSA038_uid219891/
Pseudomonas_aeruginosa_BWHPSA039_uid219892/
Pseudomonas_aeruginosa_BWHPSA040_uid219893/
Pseudomonas_aeruginosa_BWHPSA041_uid219894/
Pseudomonas_aeruginosa_BWHPSA042_uid219895/
Pseudomonas_aeruginosa_BWHPSA043_uid219896/
Pseudomonas_aeruginosa_BWHPSA044_uid219897/
Pseudomonas_aeruginosa_BWHPSA045_uid219898/
Pseudomonas_aeruginosa_BWHPSA046_uid219899/
Pseudomonas_aeruginosa_BWHPSA047_uid219900/
Pseudomonas_aeruginosa_BWHPSA048_uid219901/
Pseudomonas_aeruginosa_C20_uid219934/
Pseudomonas_aeruginosa_C23_uid219935/
Pseudomonas_aeruginosa_C3719_uid16170/
Pseudomonas_aeruginosa_C40_uid219936/
Pseudomonas_aeruginosa_C41_uid219937/
Pseudomonas_aeruginosa_C48_uid219938/
Pseudomonas_aeruginosa_C51_uid219939/
Pseudomonas_aeruginosa_C52_uid219940/
Pseudomonas_aeruginosa_CF127_uid219849/
Pseudomonas_aeruginosa_CF18_uid219850/
Pseudomonas_aeruginosa_CF27_uid219851/
Pseudomonas_aeruginosa_CF5_uid219852/
Pseudomonas_aeruginosa_CF614_uid219942/
Pseudomonas_aeruginosa_CF77_uid219941/
Pseudomonas_aeruginosa_CF_PA39_uid236781/
Pseudomonas_aeruginosa_CI27_uid89679/
Pseudomonas_aeruginosa_CIG1_uid89681/
Pseudomonas_aeruginosa_DHS01_uid224149/
Pseudomonas_aeruginosa_DHS29_uid224150/
Pseudomonas_aeruginosa_DQ8_uid170819/
Pseudomonas_aeruginosa_E2_uid219846/
Pseudomonas_aeruginosa_E2_uid89677/
Pseudomonas_aeruginosa_JJ692_uid219856/
Pseudomonas_aeruginosa_LCT_PA102_uid156843/
Pseudomonas_aeruginosa_LCT_PA220_uid208779/
Pseudomonas_aeruginosa_LCT_PA41_uid208985/
Pseudomonas_aeruginosa_M8A_1_uid219929/
Pseudomonas_aeruginosa_M8A_2_uid219930/
Pseudomonas_aeruginosa_M8A_3_uid219931/
Pseudomonas_aeruginosa_M8A_4_uid219932/
Pseudomonas_aeruginosa_M9A_1_uid219933/
Pseudomonas_aeruginosa_MH27_uid230292/
Pseudomonas_aeruginosa_MH38_uid232341/
Pseudomonas_aeruginosa_MPAO1_P1_uid80839/
Pseudomonas_aeruginosa_MPAO1_P2_uid80841/
Pseudomonas_aeruginosa_MRW44_1_uid168334/
Pseudomonas_aeruginosa_MSH10_uid219848/
Pseudomonas_aeruginosa_MSH3_uid219847/
Pseudomonas_aeruginosa_MSH_10_uid201849/
Pseudomonas_aeruginosa_N002_uid167366/
Pseudomonas_aeruginosa_NCMG1179_uid72409/
Pseudomonas_aeruginosa_PA14_uid38507/
Pseudomonas_aeruginosa_PA21_ST175_uid186902/
Pseudomonas_aeruginosa_PA45_uid189678/
Pseudomonas_aeruginosa_PABL056_uid171335/
Pseudomonas_aeruginosa_PACS2_uid16851/
Pseudomonas_aeruginosa_PADK2_CF510_uid81219/
Pseudomonas_aeruginosa_PAK_uid232360/
Pseudomonas_aeruginosa_PAK_uid66135/
Pseudomonas_aeruginosa_PAO1_CipR_uid202063/
Pseudomonas_aeruginosa_PAO1_uid201024/
Pseudomonas_aeruginosa_PAO579_uid167046/
Pseudomonas_aeruginosa_PAb1_uid28809/
Pseudomonas_aeruginosa_PFK10_uid222566/
Pseudomonas_aeruginosa_PGPR2_uid200546/
Pseudomonas_aeruginosa_PK6_uid222564/
Pseudomonas_aeruginosa_PS42_uid219943/
Pseudomonas_aeruginosa_PS50_uid219944/
Pseudomonas_aeruginosa_RB_48_uid232736/
Pseudomonas_aeruginosa_S35004_uid219860/
Pseudomonas_aeruginosa_S54485_uid219855/
Pseudomonas_aeruginosa_SG17M_uid233421/
Pseudomonas_aeruginosa_SJTD_1_uid167272/
Pseudomonas_aeruginosa_Stone_130_uid169469/
Pseudomonas_aeruginosa_U2504_uid219857/
Pseudomonas_aeruginosa_UDL_uid219854/
Pseudomonas_aeruginosa_VRFPA01_uid183455/
Pseudomonas_aeruginosa_VRFPA02_uid196397/
Pseudomonas_aeruginosa_VRFPA03_uid209587/
Pseudomonas_aeruginosa_VRFPA04_uid219238/
Pseudomonas_aeruginosa_VRFPA05_uid222642/
Pseudomonas_aeruginosa_VRFPA06_uid227489/
Pseudomonas_aeruginosa_VRFPA07_uid230365/
Pseudomonas_aeruginosa_VRFPA08_uid230869/
Pseudomonas_aeruginosa_VRFPA09_uid232744/
Pseudomonas_aeruginosa_WC55_uid202929/
Pseudomonas_aeruginosa_X13273_uid219861/
Pseudomonas_aeruginosa_X24509_uid219853/
Pseudomonas_aeruginosa_XMG_uid157339/
Pseudomonas_aeruginosa_Z61_uid219902/
Pseudomonas_aeruginosa_uid198782/
Pseudomonas_aeruginosa_uid200798/
Pseudomonas_agarici_NCPPB_2289_uid41633/
Pseudomonas_alcaligenes_MRY13_0052_uid222425/
Pseudomonas_alcaligenes_OT_69_uid202928/
Pseudomonas_alcaliphila_34_uid178463/
Pseudomonas_avellanae_BPIC_631_uid207988/
Pseudomonas_avellanae_BPIC_631_uid84293/
Pseudomonas_avellanae_CRAFRUec1_uid209401/
Pseudomonas_azotifigens_DSM_17556_uid188903/
Pseudomonas_brassicacearum_51MFCVI2_1_uid79071/
Pseudomonas_brenneri_FH4_uid186800/
Pseudomonas_caeni_DSM_24390_uid188884/
Pseudomonas_chloritidismutans_AW_1_uid182713/
Pseudomonas_chlororaphis_GP72_uid77215/
Pseudomonas_chlororaphis_O6_uid67531/
Pseudomonas_chlororaphis_YL_1_uid218608/
Pseudomonas_chlororaphis_aurantiaca_PB_St2_uid225665/
Pseudomonas_chlororaphis_aureofaciens_30_84_uid67533/
Pseudomonas_corrugata_CFBP_5454_uid196427/
Pseudomonas_cremoricolorata_DSM_17059_uid188911/
Pseudomonas_extremaustralis_14_3_substr__14_3b_uid77729/
Pseudomonas_extremorientalis_FH1_uid186799/
Pseudomonas_flectens_ATCC_12775_uid204094/
Pseudomonas_fluorescens_BBc6R8_uid20583/
Pseudomonas_fluorescens_BRIP34879_uid169520/
Pseudomonas_fluorescens_BS2_uid174875/
Pseudomonas_fluorescens_HK44_uid67805/
Pseudomonas_fluorescens_LMG_5329_uid198067/
Pseudomonas_fluorescens_NCIMB_11764_uid172562/
Pseudomonas_fluorescens_NZ007_uid41635/
Pseudomonas_fluorescens_NZ011_uid32035/
Pseudomonas_fluorescens_NZ052_uid32037/
Pseudomonas_fluorescens_NZ17_uid32039/
Pseudomonas_fluorescens_Pf29Arp_uid182080/
Pseudomonas_fluorescens_Q2_87_uid67535/
Pseudomonas_fluorescens_Q8r1_96_uid67537/
Pseudomonas_fluorescens_R124_uid68653/
Pseudomonas_fluorescens_S12_uid214469/
Pseudomonas_fluorescens_SS101_uid67539/
Pseudomonas_fluorescens_WH6_uid46613/
Pseudomonas_fluorescens_Wayne1_uid65793/
Pseudomonas_fluorescens_uid73041/
Pseudomonas_fragi_A22_uid81421/
Pseudomonas_fragi_B25_uid81403/
Pseudomonas_fuscovaginae_CB98818_uid160237/
Pseudomonas_fuscovaginae_SE_1_uid196532/
Pseudomonas_fuscovaginae_UPB0736_uid84441/
Pseudomonas_geniculata_N1_uid156719/
Pseudomonas_gingeri_NCPPB_3146_uid41405/
Pseudomonas_libanensis_FH5_uid186801/
Pseudomonas_luteola_XLDN4_9_uid170105/
Pseudomonas_mandelii_36MFCvi1_1_uid182465/
Pseudomonas_mandelii_JR_1_uid83441/
Pseudomonas_mendocina_DLHK_uid168061/
Pseudomonas_mendocina_S5_2_uid173199/
Pseudomonas_monteilii_QM_uid78179/
Pseudomonas_moraviensis_R28_uid217803/
Pseudomonas_mosselii_SJ10_uid215706/
Pseudomonas_nitroreducens_HBP1_uid232110/
Pseudomonas_oleovorans_MOIL14HWK12_uid79065/
Pseudomonas_parafulva_DSM_17004_uid188912/
Pseudomonas_pelagia_CL_AP6_uid197439/
Pseudomonas_pseudoalcaligenes_CECT_5344_uid171526/
Pseudomonas_pseudoalcaligenes_KF707_uid83639/
Pseudomonas_psychrophila_HA_4_uid170784/
Pseudomonas_psychrotolerans_L19_uid76561/
Pseudomonas_putida_B6_2_uid72885/
Pseudomonas_putida_Idaho_uid72621/
Pseudomonas_putida_LF54_uid190835/
Pseudomonas_putida_LS46_uid167222/
Pseudomonas_putida_MR3_uid195540/
Pseudomonas_putida_MTCC5279_uid176888/
Pseudomonas_putida_NB2011_uid201392/
Pseudomonas_putida_OUS82_uid223432/
Pseudomonas_putida_S11_uid171810/
Pseudomonas_putida_S12_uid171340/
Pseudomonas_putida_S12_uid222630/
Pseudomonas_putida_S13_1_2_uid214610/
Pseudomonas_putida_S610_uid218557/
Pseudomonas_putida_SJ3_uid215701/
Pseudomonas_putida_SJTE_1_uid167271/
Pseudomonas_putida_TRO1_uid174070/
Pseudomonas_putida_uid65243/
Pseudomonas_putida_uid68177/
Pseudomonas_resinovorans_DSM_21078_uid185651/
Pseudomonas_savastanoi_NCPPB_3335_uid41887/
Pseudomonas_stutzeri_ATCC_14405___CCUG_16156_uid74687/
Pseudomonas_stutzeri_B1SMN1_uid170978/
Pseudomonas_stutzeri_KOS6_uid171881/
Pseudomonas_stutzeri_MF28_uid202932/
Pseudomonas_stutzeri_NF13_uid170977/
Pseudomonas_stutzeri_SDM_LAC_uid74741/
Pseudomonas_stutzeri_T13_uid170692/
Pseudomonas_stutzeri_TS44_uid162447/
Pseudomonas_stutzeri_XLDN_R_uid168597/
Pseudomonas_synxantha_BG33R_uid67541/
Pseudomonas_syringae_1212_uid211607/
Pseudomonas_syringae_642_uid40347/
Pseudomonas_syringae_B64_uid180994/
Pseudomonas_syringae_BRIP34876_uid169408/
Pseudomonas_syringae_BRIP34881_uid169521/
Pseudomonas_syringae_BRIP39023_uid169522/
Pseudomonas_syringae_CC1416_uid211384/
Pseudomonas_syringae_CC1417_uid211390/
Pseudomonas_syringae_CC1458_uid211392/
Pseudomonas_syringae_CC1466_uid211394/
Pseudomonas_syringae_CC1513_uid211395/
Pseudomonas_syringae_CC1524_uid211397/
Pseudomonas_syringae_CC1543_uid211398/
Pseudomonas_syringae_CC1544_uid211400/
Pseudomonas_syringae_CC1559_uid211402/
Pseudomonas_syringae_CC1583_uid211404/
Pseudomonas_syringae_CC1629_uid211406/
Pseudomonas_syringae_CC1630_uid211408/
Pseudomonas_syringae_CC440_uid211409/
Pseudomonas_syringae_CC457_uid211412/
Pseudomonas_syringae_CC94_uid211414/
Pseudomonas_syringae_Cit_7_uid33213/
Pseudomonas_syringae_FF5_uid33695/
Pseudomonas_syringae_ICMP_18806_uid74973/
Pseudomonas_syringae_KCTC_12500_uid227265/
Pseudomonas_syringae_Lz4W_uid170013/
Pseudomonas_syringae_SM_uid181198/
Pseudomonas_syringae_UB246_uid211378/
Pseudomonas_syringae_UB303_uid211415/
Pseudomonas_syringae_USA007_uid211417/
Pseudomonas_syringae_USA011_uid211418/
Pseudomonas_syringae_aceris_M302273PT_uid33225/
Pseudomonas_syringae_actinidiae_CFBP_7286_uid73857/
Pseudomonas_syringae_actinidiae_CH2010_6_uid75359/
Pseudomonas_syringae_actinidiae_ICMP_18708_uid74965/
Pseudomonas_syringae_actinidiae_ICMP_18744_uid74967/
Pseudomonas_syringae_actinidiae_ICMP_18800_uid74969/
Pseudomonas_syringae_actinidiae_ICMP_18801_uid167321/
Pseudomonas_syringae_actinidiae_ICMP_18804_uid167425/
Pseudomonas_syringae_actinidiae_ICMP_18804_uid74971/
Pseudomonas_syringae_actinidiae_ICMP_18807_uid167414/
Pseudomonas_syringae_actinidiae_ICMP_18807_uid178798/
Pseudomonas_syringae_actinidiae_ICMP_18883_uid167413/
Pseudomonas_syringae_actinidiae_ICMP_18886_uid165719/
Pseudomonas_syringae_actinidiae_ICMP_19068_uid167433/
Pseudomonas_syringae_actinidiae_ICMP_19070_uid167432/
Pseudomonas_syringae_actinidiae_ICMP_19071_uid167435/
Pseudomonas_syringae_actinidiae_ICMP_19072_uid165721/
Pseudomonas_syringae_actinidiae_ICMP_19073_uid167434/
Pseudomonas_syringae_actinidiae_ICMP_19079_uid167408/
Pseudomonas_syringae_actinidiae_ICMP_19094_uid167411/
Pseudomonas_syringae_actinidiae_ICMP_19095_uid167412/
Pseudomonas_syringae_actinidiae_ICMP_19096_uid167415/
Pseudomonas_syringae_actinidiae_ICMP_19097_uid167406/
Pseudomonas_syringae_actinidiae_ICMP_19098_uid167416/
Pseudomonas_syringae_actinidiae_ICMP_19099_uid167422/
Pseudomonas_syringae_actinidiae_ICMP_19100_uid167423/
Pseudomonas_syringae_actinidiae_ICMP_19101_uid167407/
Pseudomonas_syringae_actinidiae_ICMP_19102_uid167428/
Pseudomonas_syringae_actinidiae_ICMP_19103_uid167430/
Pseudomonas_syringae_actinidiae_ICMP_19104_uid167431/
Pseudomonas_syringae_actinidiae_ICMP_19439_uid179101/
Pseudomonas_syringae_actinidiae_ICMP_19455_uid178611/
Pseudomonas_syringae_actinidiae_ICMP_9853_uid74963/
Pseudomonas_syringae_actinidiae_ICMP_9855_uid167427/
Pseudomonas_syringae_actinidiae_KW41_uid73861/
Pseudomonas_syringae_actinidiae_M303091_uid33217/
Pseudomonas_syringae_actinidiae_PA459_uid73865/
Pseudomonas_syringae_actinidiae_Shaanxi_M228_uid76165/
Pseudomonas_syringae_actinidiae_Shaanxi_M7_uid76167/
Pseudomonas_syringae_actinidiae_TP1_uid74975/
Pseudomonas_syringae_actinidiae_TP6_1_uid74977/
Pseudomonas_syringae_actinidiae_uid68657/
Pseudomonas_syringae_actinidiae_uid68659/
Pseudomonas_syringae_actinidiae_uid68663/
Pseudomonas_syringae_aesculi_0893_23_uid33201/
Pseudomonas_syringae_aesculi_2250_uid39921/
Pseudomonas_syringae_aesculi_NCPPB3681_uid39839/
Pseudomonas_syringae_aptata_DSM_50252_uid33221/
Pseudomonas_syringae_atrofaciens_DSM_50255_uid214371/
Pseudomonas_syringae_avellanae_ISPaVe013_uid84319/
Pseudomonas_syringae_avellanae_ISPaVe037_uid84321/
Pseudomonas_syringae_glycinea_B076_uid51517/
Pseudomonas_syringae_glycinea_R4_uid33197/
Pseudomonas_syringae_glycinea_race_4_uid51519/
Pseudomonas_syringae_japonica_M301072PT_uid33209/
Pseudomonas_syringae_lachrymans_M301315_uid33205/
Pseudomonas_syringae_lachrymans_M302278PT_uid33219/
Pseudomonas_syringae_maculicola_ES4326_uid33215/
Pseudomonas_syringae_mori_301020_uid33207/
Pseudomonas_syringae_morsprunorum_M302280PT_uid33203/
Pseudomonas_syringae_oryzae_1_6_uid31357/
Pseudomonas_syringae_panici_LMG_2367_uid160239/
Pseudomonas_syringae_phaseolicola_1644R_uid72523/
Pseudomonas_syringae_pisi_1704B_uid33211/
Pseudomonas_syringae_pisi_H5E3_uid211606/
Pseudomonas_syringae_tabaci_6605_uid165717/
Pseudomonas_syringae_tabaci_ATCC_11528_uid32261/
Pseudomonas_syringae_tabaci_ATCC_11528_uid38647/
Pseudomonas_syringae_theae_ICMP_3923_uid168431/
Pseudomonas_syringae_theae_NCPPB_2598_uid208000/
Pseudomonas_syringae_theae_NCPPB_2598_uid73855/
Pseudomonas_syringae_tomato_K40_uid40341/
Pseudomonas_syringae_tomato_Max13_uid40343/
Pseudomonas_syringae_tomato_NCPPB_1108_uid40345/
Pseudomonas_syringae_tomato_T1_uid19697/
Pseudomonas_syringae_uid178390/
Pseudomonas_taeanensis_MS_3_uid217843/
Pseudomonas_taiwanensis_DSM_21245_uid188913/
Pseudomonas_taiwanensis_SJ9_uid215705/
Pseudomonas_thermotolerans_DSM_14292_uid187118/
Pseudomonas_thermotolerans_J53_uid210336/
Pseudomonas_tolaasii_6264_uid89427/
Pseudomonas_tolaasii_NCPPB_2192_uid32041/
Pseudomonas_tolaasii_PMS117_uid66663/
Pseudomonas_umsongensis_20MFCvi1_1_uid178050/
Pseudomonas_veronii_1YdBTEX2_uid189715/
Pseudomonas_viridiflava_CC1582_uid211420/
Pseudomonas_viridiflava_TA043_uid211421/
Pseudomonas_viridiflava_UASWS0038_uid19911/
Pseudomonas_vranovensis_DSM_16006_uid188914/
Psychrobacter_1501_2011__uid65121/
Psychrobacter_AC24_uid228289/
Psychrobacter_PAMC_21119_uid76621/
Psychrobacter_TB2_uid228349/
Psychrobacter_TB47_uid228382/
Psychrobacter_TB67_uid228384/
Psychrobacter_TB_15_uid228323/
Psychrobacter_aquaticus_CMS_56_uid210528/
Psychrobacter_lutiphocae_DSM_21542_uid187093/
Rhizobacter_JGI_0001007_A16_uid190858/
Rhizobacter_JGI_0001009_E21_uid190857/

## References

1. Hyatt D, Chen G-L, LoCascio PF, Land ML, Larimer FW, Hauser LJ. Prodigal: Prokaryotic gene recognition and translation initiation site identification. BMC Bioinformatics. 2010;11. doi:[10.1186/1471-2105-11-119](https://doi.org/10.1186/1471-2105-11-119)

2. Larsen TS, Krogh A. EasyGene - a prokaryotic gene finder that ranks ORFs by statistical significance. BMC Bioinformatics. 2003;4. doi:[10.1186/1471-2105-4-21](https://doi.org/10.1186/1471-2105-4-21)

3. Moreno-Hagelsieb G, Latimer K. Choosing BLAST options for better detection of orthologs as reciprocal best hits. Bioinformatics Advance Access. 2007; doi:[10.1093/bioinformatics/btm585](https://doi.org/10.1093/bioinformatics/btm585)

4. Bailey TL, Williams N, Misleh C, Li WW. MEME: Discovering and analyzing DNA and protein sequence motifs. Nucleic Acids Res. 2006;34: W369–73. doi:[10.1093/nar/gkl198](https://doi.org/10.1093/nar/gkl198)

1. A.k.a Perl and Bash scripts. <http://xkcd.com/224/> [↑](#footnote-ref-28)
