# Supplementary material for: The ECF sigma factor, PSPTO_1043, in Pseudomonas syringae pv. tomato DC3000 is induced by oxidative stress and regulates genes involved in oxidative stress response
Source: PLoS One. 2017 Jul 12;12(7):e0180340. doi: 10.1371/journal.pone.0180340 (PMC5507510; doi:10.1371/journal.pone.0180340)
Supplement: S2 Text — (DOCX) [file pone.0180340.s005.docx]

A comparison of consensus sequences

Paul Stodghill

## Introducion

In Figure 1 of the paper, “The ECF sigma factor, PSPTO_1043, in *Pseudomonas* *syringae* pv. *tomato* DC3000 is induced by oxidative stress and regulates genes involved in oxidative stress and virulence” by Butcher, et al., we present a putative motif for the ECF sigma factor, PSPTO_1043, as identified by MEME [1]. The motif is shown here in Figure Figure 1. We assert in the paper that the motif “closely resembles the RpoE${}_{mboxRsp}$-controlled promoter region identified in *Rhodobacter* and *Caulobacter crescentus*”. This document provides evidence of this claim.


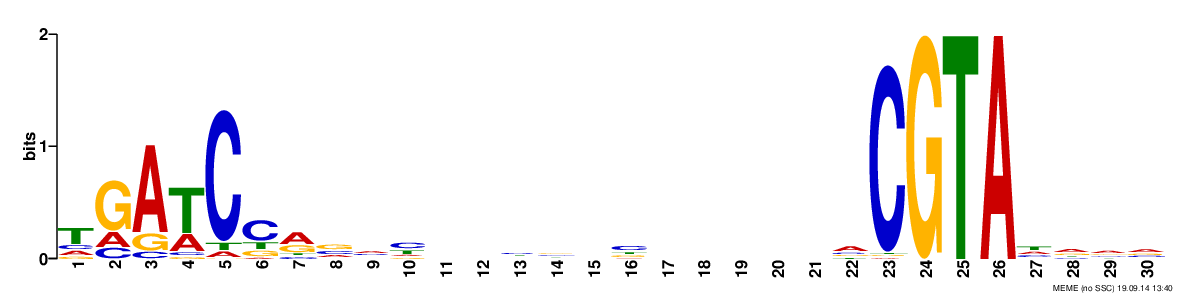


Figure 1: Putative 1043 promoter motif

The methods used to construct the motifs in our paper and the other two papers is very different. Furthermore, the *Rhodobacter* and *Caulobacter* motifs were constructed to allow variable spacing between the -35 and -10 boxes, and ours was not. For these reasons, a direct comparison between the three motifs is very difficult.

Instead, we have chosen to reconstruct the motif from each of the three organisms using the same method, present the motifs, and allow the reader to decide if the evidence supports our claim.

## Method

Our method is as follows. First, the sequences that were used to construct each of *P. syringae*, *Rhodobacter*, and *Caulobacter* motifs were gathered. For *P. syringae*, the sequences were extracted from first motif identified MEME [1], as described in our paper; these sequence appear below under the heading “Sequences from *Pseudomonas syringae*” For *Rhodobacter*, the sequences were extracted from the six previously identified promoters (bold) and three experimentally confirmed promoters (starred) from Table 1 in [2]; these sequence appear below under the heading “Sequences from *Rhodobacter sphaeroides*”. For *Caulobacter*, the sequences were extracted from Figure 6A in [3]; these sequence appear below under the heading “Sequences from *Caulobacter crescentus*”.

Second, each of the three sets of sequences were used as input of GLAM2 [4] version 4.10.2, a program for discovering motifs and consensus sequences that allows insertions and deletions. The following command line was used for each run of GLAM2.

glam2 -J 5.0 -a 25 -b 35 -n 10000
 -o output_data n input_sequences.fna

## Results

The first motif for each of the different organisms is show in Figures Figure **¿fig:glam2_psyr?**, Figure **¿fig:glam2_rhodo?**, and Figure **¿fig:glam2_caul?**.


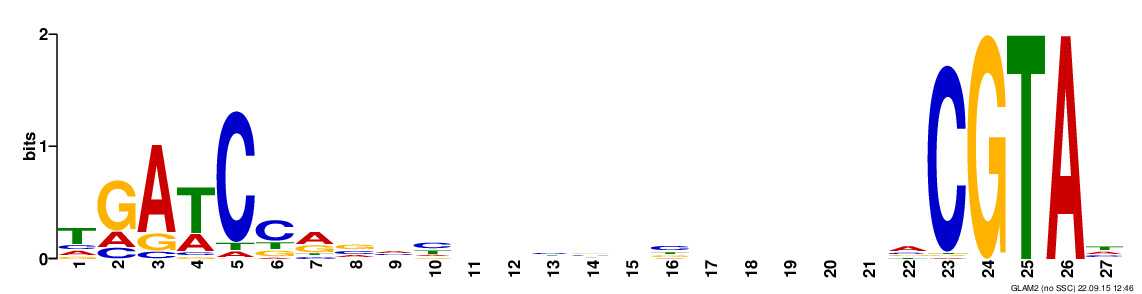
 {#fig:glam2_psyr}


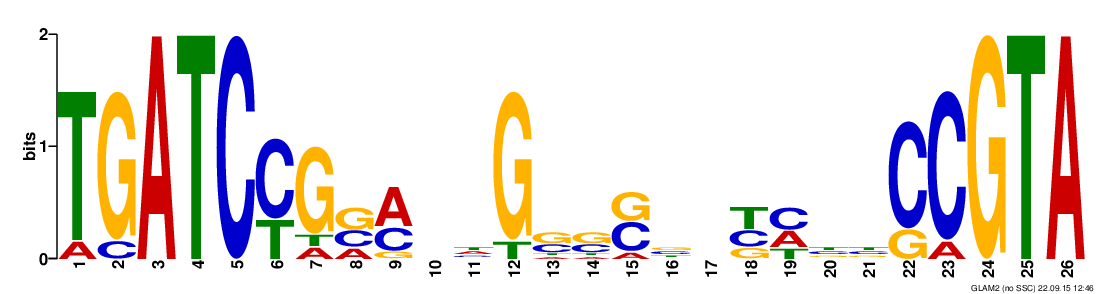
 {#fig:glam2_rhodo}


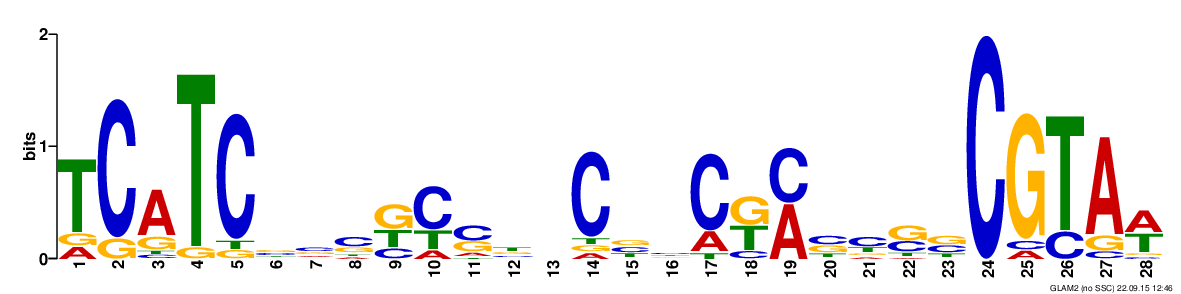
 {#fig:glam2_caul}

## Discussion

The *P. syringae* motif produced GLAM2 (Figure Figure **¿fig:glam2_psyr?**) is virtually identical to the motif produced MEME (Figure Figure 1). The *Rhodobacter* and *Caulobacter* motifs are similar to those that appear in [2] and [3], respectively.

The three motifs produced by GLAM2 are similar, but not identical. The -35 box of the *P. syringae* and *Rhodobacter* motifs are very similar. The -10 box of the *P. syringae* and *Caulobacter* motifs are also very similar.

## Sequences from *Pseudomonas syringae*

For *P. syringae*, the sequences were extracted from first motif identified MEME [1], as described in our paper.

>1937070-1937170/NC_004578
TGATCCAGACCGCCAGTGCCGACGTATTGC
>4886439-4886539/NC_004578
TGATCTAGGCTGTGTTTACCAACGTACTAA
>2076746-2076846/NC_004578
TGATCCGCATCTTTACGAAACACGTACATC
>459085-459185/NC_004578
CGATCCACCTGCGTTCATCAAACGTAAAAA
>1142635-1142735/NC_004578
TGATCCACTCTTCATCCCGCTACGTAACAC
>4405940-4406040/NC_004578
TGATCCAAAGCATGGCTGCTATCGTAAGCA
>5483368-5483468/NC_004578
TGATCCACCTGCCCGCAAGCAACGTAGCGG
>916172-916272/NC_004578
TGAACCATGTTAACGCTAGCGCCGTATAGA
>2861563-2861663/NC_004578
TGATCCAGTGTGCGCCTGCCTGCGTATGTT
>1526652-1526752/NC_004578
TGATCCCGACAGTGTGCGCGGACGTATTCA
>2138680-2138780/NC_004578
TGATCAGGATGCCCTTGATCGTCGTATCGA
>5093220-5093320/NC_004578
CGAACCGGACGCTGCGCAACTGCGTAAACA
>3247623-3247723/NC_004578
GGATCCAAACTGCAAATTTATTCGTATGTA
>3550401-3550501/NC_004578
CGATCGACATCAGGCTCTGCGACGTAAAAT
>6244351-6244451/NC_004578
TGATCGGAATAGTGCCGGACCACGTAACGC
>4801414-4801514/NC_004578
TAATCCAGCCCATCGGCCCGCCCGTATTTA
>790923-791023/NC_004578
CGATCGACACGGCTCATCCATTCGTATGCA
>6249884-6249984/NC_004578
CGATCCACCGAGCCGCCCAGCACGTAAACG
>4188631-4188731/NC_004578
TGAACTGGCCGAGCACGAAGTCCGTATGAC
>3018341-3018441/NC_004578
CAATCCCGGTTTCCGCTTTTGACGTATTCA
>1190219-1190319/NC_004578
TGATCCAAAAGGGCCGCTGTTCCGTACGAT
>4360375-4360475/NC_004578
TCATCCGCAACATGGAGTCTGCCGTATGAG
>6011819-6011919/NC_004578
TGGTCAGGTCCTATGGGCTCGACGTATTAA
>2906277-2906377/NC_004578
TGATCCCTGCCTATACAACATACGTATGTC
>5349873-5349973/NC_004578
GAAACGACATTGAGTCTTTTTTCGTACAAA
>4800032-4800132/NC_004578
AAATCGAGACGGGTTGCATCACCGTAAAAC
>420298-420398/NC_004578
CCAACCAGACTTCATCCTCGTGCGTATCAA
>3585137-3585237/NC_004578
TGATCGGCAACGATCTGGCGAACGTACTGG
>261116-261216/NC_004578
TCATCGCGACGCTTACGATCTGCGTAAGAC
>4204843-4204943/NC_004578
TGATCCTGCTGCTCGGCCAGTCCGTAACGG
>3321840-3321940/NC_004578
AGAACCTCGACATCGTCATAAACGTAAATA
>5437487-5437587/NC_004578
TGATACAGCCCGAAGGCGGCCACGTACAAC
>3117255-3117355/NC_004578
AGCTCCAAAAACTACTGTTATACGTACAAG
>6220133-6220233/NC_004578
TGAACTGACTGTCGTTACCCACCGTAAGGA
>2529216-2529316/NC_004578
TCATCTCAAGACCTGTGCATGACGTAAAAG
>5278411-5278511/NC_004578
CAAACTAGGTGATCTCGATCTTCGTAGAAA
>2480413-2480513/NC_004578
GGATCGTCACCGCTGCTGTGACCGTATACG
>2274677-2274777/NC_004578
TGAACTCGCCGTGCTCGTAAACCGTATCCA
>560882-560982/NC_004578
TAAACCTGCTTACACTGATCTGCGTATCGG
>639417-639517/NC_004578
GGATCTGGAATACGGATACCTTCGTAGTAA
>1235570-1235670/NC_004578
AGATCCATAACGCCGAGCTGCTCGTACAGG
>5115755-5115855/NC_004578
TAATCTGATGATCGCTGTGCGACGTATCTG
>335391-335491/NC_004578
TGATACAACTCGCGTGTCGGATCGTAAGAC
>1069854-1069954/NC_004578
TGATCTAATTTCCACCATACTCCGTAGTCT
>5189496-5189596/NC_004578
TGATCGGCCTATAAAGACAGCTCGTATAGA
>1790970-1791070/NC_004578
TGATCAGACAGATCGACCGCTACGTAAGGG
>994790-994890/NC_004578
TGAACCATGTCCAGCGGGATGACGTAGGTG
>320023-320123/NC_004578
GAAACGACACACTGTGTTGAAACGTACACA
>4934466-4934566/NC_004578
AAACCCATCTCGTTACCTTTTACGTAAGAC
>3178935-3179035/NC_004578
TCGTCCGCCTGAGCCAGTTCTTCGTACAAC
>2867765-2867865/NC_004578
TGAGCCAATATTGACTCAAAGCCGTACAAA
>40804-40904/NC_004578
TGATCCAATGTCCCTCCAACGAGGTATATC
>5698692-5698792/NC_004578
TGGTCCTGCTTGAGGGCCAGTGCGTAAGCG
>4875681-4875781/NC_004578
GGATCTCAACCTGGCCATCTACCGTATCCA
>1895320-1895420/NC_004578
TGATCTTGTCAGATTCAGCAGGCGTACTTC
>4765198-4765298/NC_004578
GCATCCGGGCGCCTGTTTTACACGTACCCG
>4388075-4388175/NC_004578
CAATCTGGCCATCCACCTGAACCGTACGGC
>1423979-1424079/NC_004578
CAAACCAGGCATCAGCTTCAGCCGTATCCT
>3204078-3204178/NC_004578
CAAACCATAAGCATTCTCAACTCGTACTAA
>1315733-1315833/NC_004578
TGATGCGATCAATACCCCGTAACGTAAGAA
>1218967-1219067/NC_004578
TCATCCACCAGAACAGGGGAGCCGTAGATG
>5329019-5329119/NC_004578
AACTCTACCCCTACCGACTTTTCGTACAAA
>6256614-6256714/NC_004578
TGACCCGGTTACCAGCGAGTGGCGTAAGGT
>3885151-3885251/NC_004578
TGGATCAGATCAGCCGTTTGTACGTACGTC
>4030913-4031013/NC_004578
AGCACCACTCTGAGCGGTTCTGCGTACAGT
>2060616-2060716/NC_004578
GCAACCAGAACCGGTTGCATAGCGTAGAAC
>1021416-1021516/NC_004578
AAACCGACCCGACCCTCACCAGCGTATTGA
>5318883-5318983/NC_004578
TGGGTCGGCCGCTTGAGCCTGACGTATAAA
>1504123-1504223/NC_004578
TGACCTCTTGATGCGCTTGCGACGTATAAC
>6186939-6187039/NC_004578
GCGTCTGCCTGATGGTGGTCAGCGTAAATA
>6152307-6152407/NC_004578
AGGTCGAGTTTTCCATTACCCGCGTATCTT
>239166-239266/NC_004578
TCGTTCTGGCCAATACAGCGTACGTATAAA
>4422244-4422344/NC_004578
GAACCAGGCGTGCGTCCAATAACGTATAAG
>4683422-4683522/NC_004578
AGGTCACCACTTCGCAGTTGTACGTATCTT
>2735369-2735469/NC_004578
ACATTCAAGACCTGACAAAACACGTAAAGG
>132689-132789/NC_004578
TCCTCCATGCCCATGATGAACACGTACGGA
>3678273-3678373/NC_004578
TGAGCTGATTCAGGATGAGCGTCGTACGAT
>2695532-2695632/NC_004578
TAAAAGCCACGCACCTTTCAGGCGTAAAAC
>57150-57250/NC_004578
CAGACCGCACTGCTCCGGGTCGCGTATTCT
>3288413-3288513/NC_004578
CCGATCAGAGCGCCGCAATTGACGTACAGG
>3029145-3029245/NC_004578
CGGTCCTCGATGATCTTCTGATCGTAATCG
>609210-609310/NC_004578
TGATCGGTTTCCAGGTCCTGGAAGTAATAC
>1296499-1296599/NC_004578
AGATAATGCCATCGACAGCTCACGTATCAG
>2037704-2037804/NC_004578
AGCACGAATGCCTTGGTGGCACCGTAAACA
>1507779-1507879/NC_004578
AGCACTGCGCTGTTCAAACTTCCGTAGAAC
>757587-757687/NC_004578
CGGTCCTGACTGCTGGGCAAAATGTAGGTA
>497044-497144/NC_004578
AAGGCTGACGATGGCCGTAAGGCGTATAAG

## Sequences from *Rhodobacter sphaeroides*

For *Rhodobacter*, the sequences were extracted from the six previously identified promoters (bold) and three experimentally confirmed promoters (starred) from Table 1 in [2].

>RSP1087-1091
TGATCCGccttgggcgacagTCCGTAT
>RSP0601
TGATCCGgacatgtgtttttTCCGTAG
>RSP2143-2144
TGATCCGggaagcgggcccgCGCGTAA
>RSP1092-1093
TGATCCAgactggcccggccGCCGTAA
>RSP1409
TCATCCGccggagccgccttcTGCGTAG
>RSP1852
TGATCTGaaccgtcgcttaaCCCGTAT
>RSP0296
TGATCCGgaacgcgcggcccGCAGTAG
>RSP6222
TGATCTTcatggggatatctCCCGTAG
>RSP3336
AGATCTGacgtgaacaagatACCGTAA

## Sequences from *Caulobacter crescentus*

For *Caulobacter*, the sequences were extracted from Figure 6A in [3].

>cc0229
TCATTttcgcccccttacaagtCGTAA
>cc0351
ACATCcgcgtcgacggcgttcgCGTAT
>cc0353
TCGTCatgtcgtttcccttttcCGTAG
>cc0358
TGATCgcctcggacgcctactggCGCAA
>cc0417
TCATCatgggcctgaacctgacCGAGT
>cc0419
ACAGCgccccctccgtctcgccgCGTAT
>cc0459
GCGTCtaagtttccggcccggcCGTAT
>cc0462
TCATGggcatgctgctgcggttCGTCA
>cc0648
TCATCcgttcccgccaaggctcCGTAT
>cc1160
TCTTCcggctgatcaccgcgcgcCGCAA
>cc1428
TGATCcacgacggctattccagCGTAT
>cc1625
TCATCaagccataccgcgcgcgCATGA
>cc2392
TCCTCgcatcgtggttcggcgaCCTAT
>cc2575
TCATCgccgtagccttctttctCGTCC
>cc2600
GCGTCcgttcgtcacgatgacgCGTAA
>cc2806
TCATGgccgacctcgccgccgcCGTGA
>cc2916
TCATCtcggccagccccgaccgtCGCAA

## References

1. Bailey TL, Williams N, Misleh C, Li WW. MEME: Discovering and analyzing DNA and protein sequence motifs. Nucleic Acids Res. 2006;34: W369–73. doi:[10.1093/nar/gkl198](https://doi.org/10.1093/nar/gkl198)

2. Dufour YS, Landick R, Donohue TJ. Organization and evolution of the biological response to singlet oxygen stress. J Mol Biol. 2008;383: 713–730. doi:[10.1016/j.jmb.2008.08.017](https://doi.org/10.1016/j.jmb.2008.08.017)

3. Lourenço RF, Gomes SL. The transcriptional response to cadmium, organic hydroperoxide, singlet oxygen and UV-A mediated by the $\sigma^{E}$-ChrR system in *Caulobacter crescentus*. Mol Microbiol. 2009;72: 1159–1170. doi:[10.1111/j.1365-2958.2009.06714.x](https://doi.org/10.1111/j.1365-2958.2009.06714.x)

4. Frith MC, Saunders NFW, Kobe B, Bailey TL. Discovering sequence motifs with arbitrary insertions and deletions. PLoS Comput Biol. 2008;4: e1000071. doi:[10.1371/journal.pcbi.1000071](https://doi.org/10.1371/journal.pcbi.1000071)
