# Supplementary material for: The ECF sigma factor, PSPTO_1043, in Pseudomonas syringae pv. tomato DC3000 is induced by oxidative stress and regulates genes involved in oxidative stress response
Source: PLoS One. 2017 Jul 12;12(7):e0180340. doi: 10.1371/journal.pone.0180340 (PMC5507510; doi:10.1371/journal.pone.0180340)
Supplement: S3 Text — (DOCX) [file pone.0180340.s006.docx]

Effect of singlet O_2_ and tBOOH on Wildtype and ΔPSPTO_1043/1042 *Pseudomonas syringae* pv *tomato* DC3000

Bryan Swingle, Melanie Filiatrault, Zhongmeng Bao, Paul Stodghill

## Introduction

Experiments were performed to assess whether or not singlet O_2_ and tert-Butyl hydroperoxide (tBOOH) alter light emission of lux fusions in strains used in the main paper, namely wildtype *Pseudomonas syringae* pv *tomato* DC3000 (WT) and a PSPTO_1043/1042 double mutant (ΔPSPTO_1043/1042). In order to test this hypothesis, a lux-fusion using the promoter of the housekeeping gene, *gap1*, was constructed and transformed into the two target strains by electroporation.

## *lux* assays

The *gap1* promoter (Pgap1) was cloned using the primers shown in Table 1. The Pgap1::lux vector (pBS70) was constructed by LR recombination the destination vector pBS59 ([1]) and entry vector pBS66. The entry vector (pBS66) was made by Topo cloning into pENTR/D the PCR product of oSWC485 and oSWC486, which amplifed a 286 bp region from the *Pseudomonas syringae* pv *tomato* DC3000 genome. The amplified 286 bp region spanned the intergenic region between the *gap1* and *edd* genes. This fragment also included the first 9 codons of the gap1 gene and the first 7 codons of the divergently transcribed *edd* gene. All vectors were confirmed by sequence analysis. The pBS70 plasmid was transformed into wildtype DC3000 and the PSPTO_1043/1042 double mutant by electroporation.

Table 1: Primers used to construct pBS70

| Primer | Locus | Sequence |
| --- | --- | --- |
| oSWC485 | 5’ *gap-1* intergenic (includes CACC) | CACCCTTCAAGGACGCGGGGATGCATGTG |
| oSWC486 | 3’ *gap-1* intergenic | ACCGTTGATTGCGATACGGAGAGTC |

Lux expression assays of the two strains in the presence of singlet O_2_ and tBOOH were conducted using the method described in the main paper.

Figure 1 shows the lux expression values from 0 to 6 hours of the WT and ΔPSPTO_1043/1042 strains grown with 2.5$mu$M Rose Bengal in both “dark” (no singlet O_2_) and “light” (singlet O_2_) conditions. Figure 2 shows the lux expression values from 0 to hours of the WT and ΔPSPTO_1043/1042 strains grown with no and 0.1mH tBOOH.

Figure 1: 2.5μM Rose Bengal, light off and on

Figure 2: 0mM and 0.1mM tBOOH

The curves for both strain under the control and test conditions are essentially the same. The presence of singlet O_2_ and tBOOH in the concentrations used for the main paper does not appear to effect the transcriptional activity of the housekeeping gene, *gap1*. From this, we deduce that the differences observed in expression of the PSPTO_1043 regulon genes is attributable to something else. The evidence presented in the main paper suggests that it is the presence or absence of PSPTO_1043/1042.

## Growth assays

Experiments were performed to assess whether or not the presence of Rose Bengal alters growth of strains used in the main paper, namely wild type *Pseudomonas* *syringae* pv *tomato DC3000* (WT) and a PSPTO_1043/1042 double mutant (ΔPSPTO_1043/1042). In order to test this strains were grown in the light (Figure 3) and the dark (Figure 4) with various concentrations of Rose Bengal. A representative growth curve is presented for conditions with light and conditions in the dark. The results show that no differences in growth are observed between the wild-type and mutant strains. The experiment was repeated three times and similar results were observed.

Figure 3: Growth in singlet O_2_ in light

Figure 4: Growth in singlet O_2_ in dark

### References

1. Swingle B, Thete D, Moll M, Myers CR, Schneider DJ, Cartinhour S. Characterization of the PvdS-regulated promoter motif in *Pseudomonas syringae* pv. *tomato* DC3000 reveals regulon members and insights regarding PvdS function in other pseudomonads. Mol Microbiol. 2008;68: 871–89. doi:[10.1111/j.1365-2958.2008.06209.x](https://doi.org/10.1111/j.1365-2958.2008.06209.x)
