# Supplementary material for: The ECF sigma factor, PSPTO_1043, in Pseudomonas syringae pv. tomato DC3000 is induced by oxidative stress and regulates genes involved in oxidative stress response
Source: PLoS One. 2017 Jul 12;12(7):e0180340. doi: 10.1371/journal.pone.0180340 (PMC5507510; doi:10.1371/journal.pone.0180340)
Supplement: S4 Text — (PDF) [file pone.0180340.s007.pdf]

Assays to measure growth of wildtype and ΔPSPTO_1043/1042 *Pseudomonas syringae* pv *tomato* DC3000 in *Arabidopsis* seedlings.

Bronwyn Butcher Melanie Filiatrault Paul Stodghill

Singlet oxygen ($^1O_2$) is produced as part of the plant defense system during plant pathogen interactions that can be directly antimicrobial or potentiate the antimicrobial affects of other plant generated antimicrobial substances (reviewed in [1]). We hypothesized that PSPTO_1043/1042 may play a role in the colonization of plants by DC3000 by providing functions that counteract the $^1O_2$ associated plant defense responses. We tested WT and the ΔPSPTO_1043/1042 double mutant for growth in *Arabidopsis* seedlings using the flooding method described in [2]. The results are shown in Figure 1.

We detected a small, but reproducible and statistically significant (two-tailed t-test, $p < 0.05$), decrease in bacterial growth of the ΔPSPTO_1043/1042 double mutant at the early stages of infection (2 days post infection (PI)), but the difference was no longer apprarent 4 days PI. These results suggest that PSPTO_1043/1042 might be important at early stages of infection or colonization and that the requirement for these genes or their regulated functions declines at later stages of infection.

It should be noted that during these experiments the *Arabidopsis* seedlings were exposed to a suspension of cells at a higher concentration than are likely to occur naturally. Therefore, while the effect of a PSPTO_1043/1042 deletion was only slight in our experiments, it is possible that PSPTO_1043/1042 is important in allowing DC3000 to evade the plant's defenses in early stages of infection or colonization and ultimately proliferate when only a few cells enter the plant, as is the case during a natural infection. Addition experiments would be needed to test this hypothesis.

References

[1] Triantaphylidès C, Havaux M. Singlet oxygen in plants: production, detoxification and signaling. Trends Plant Sci. 2009;14(4):219–228. doi:10.1016/j.tplants.2009.01.008.

[2] Park SH, Butcher BG, Anderson Z, Pellegrini N, Bao Z, D'Amico K, et al. Analysis of the small RNA *P16/RgsA* in the plant pathogen *Pseudomonas syringae pv.* tomato strain DC3000. Microbiology. 2013;159(2):296–306. doi:10.1099/mic.0.063826-0.

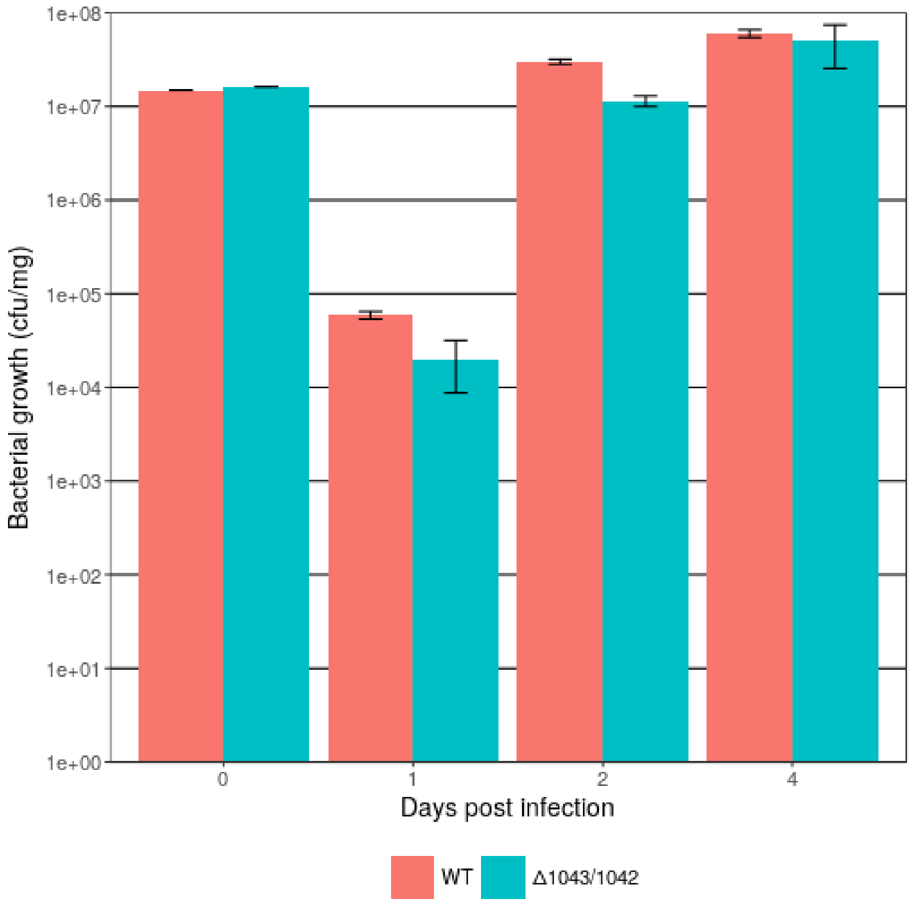

Figure 1: **Growth of WT DC3000 and PSPTO_1043/1042 in *Arabidopsis* seedlings.** WT DC3000 (salmon) and ΔPSPTO_1043/1042 deletion mutant (turquoise) strains were used to infect *Arabidopsis* seedlings. Growth of the strains within the plant was determined 1, 2 and 4 days post infection.
